# 4,2’:6’,4”- and 3,2’:6’,3”-Terpyridines: The Conflict between Well-Defined Vectorial Properties and Serendipity in the Assembly of 1D-, 2D- and 3D-Architectures

**DOI:** 10.3390/ma10070728

**Published:** 2017-06-30

**Authors:** Y. Maximilian Klein, Alessandro Prescimone, Edwin C. Constable, Catherine E. Housecroft

**Affiliations:** Department of Chemistry, University of Basel, Spitalstrasse 51, 4056 Basel, Switzerland; max.klein@unibas.ch (Y.M.K.); alessandro.prescimone@unibas.ch (A.P.); edwin.constable@unibas.ch (E.C.C.)

**Keywords:** coordination polymer, coordination network, 4,2’:6’,4”-terpyridine, cobalt thiocyanate

## Abstract

A comparative investigation of the coordination assemblies formed between Co(NCS)_2_ and two monotopic 4,2’:6’,4’’-terpyridine (4,2’:6’,4”-tpy) ligands or two related ditopic ligands is reported. Crystals were grown by layering MeOH solutions of Co(NCS)_2_ over a CHCl_3_ or 1,2-C_6_H_4_Cl_2_ solution of the respective ligand at room temperature. With 4’-(2-methylpyrimidin-5-yl)-4,2’:6’,4”-terpyridine (**6**), the 1D-coordination polymer {[Co_2_(NCS)_4_(MeOH)_4_(**6**)_2_]∙2MeOH∙8H_2_O}*_n_* assembles with **6** coordinating only through the outer N-donors of the 4,2’:6’,4”-tpy unit; coordination by the MeOH solvent blocks two cobalt coordination sites preventing propagation in a higher-dimensional network. A combination of Co(NCS)_2_ and 1-(4,2‘:6’,4”-terpyridin-4’-yl)ferrocene (**7**) leads to {[Co(NCS)_2_(**7**)_2_]∙4CHCl_3_}*_n_* which contains a (4,4) net; the 2D-sheets associate through π-stacking interactions between ferrocenyl and pyridyl units. A 3D-framework is achieved through use of the ditopic ligand 1,4-bis(*^n^*propoxy)-2,5-bis(4,2’:6’,4”-terpyridin-4’-yl)benzene (**8**) which acts as a 4-connecting node in {[Co(NCS)_2_(**8**)_2_]∙2C_6_H_4_Cl_2_}*_n_*; the combination of metal and ligand planar 4-connecting nodes results in a {6^5^.8} **cds** net. For a comparison with the coordinating abilities of the previously reported 1,4-bis(*^n^*octoxy)-2,5-bis(4,2’:6’,4”-terpyridin-4’-yl)benzene (**3**), a more flexible analogue **9** was prepared. {[Co(NCS)_2_(**9**)]∙2CHCl_3_}*_n_* contains a (4,4) net defined by both metal and ligand planar 4-connecting nodes. The *^n^*octoxy tails of **9** protrude from each side of the (4,4) net and thread through adjacent sheets; the arene-attached *^n^*octoxy chains associate through a combination of van der Waals and C–H...π interactions.

## 1. Introduction

**Scheme 1:**
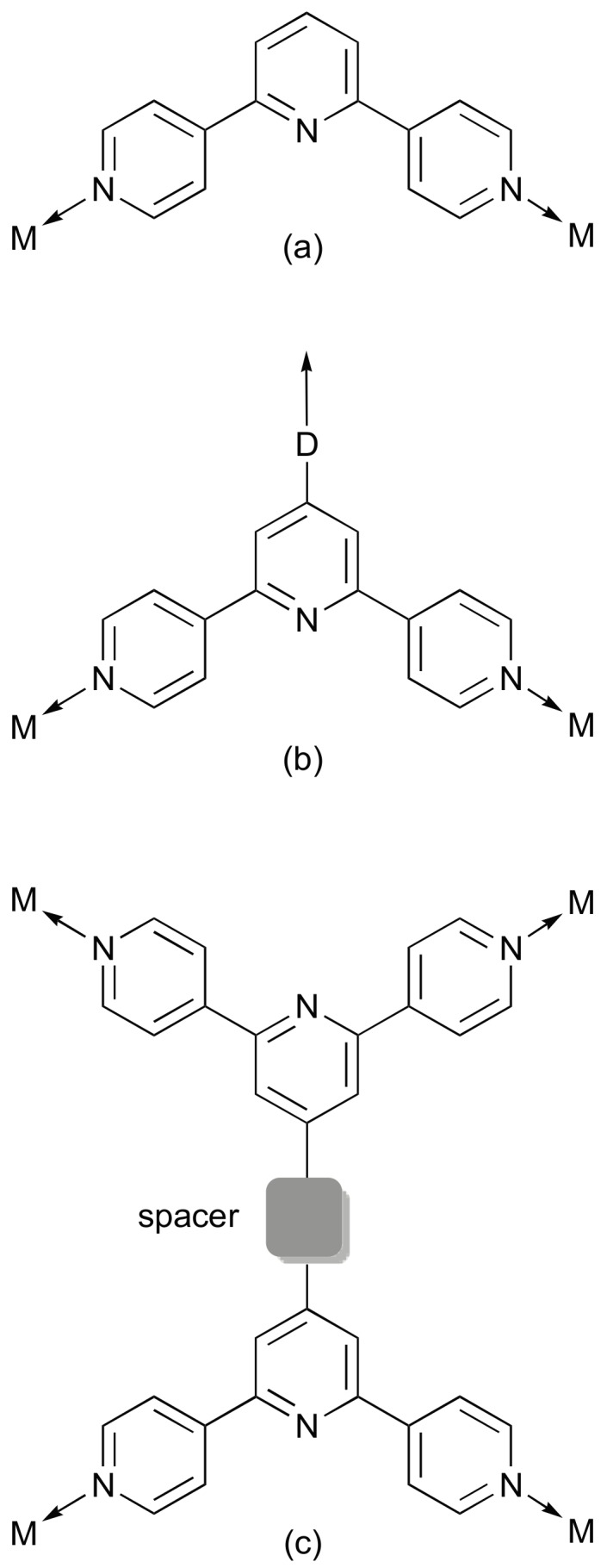
(**a**) Divergent V-shaped motif of 4,2’:6’,4”-tpy; (**b**) introduction of a donor group, D, in the 4’-position; and (**c**) 4-connecting node presented by ditopic bis(4,2’:6’,4”-tpy).

The coordination chemistry of divergent 4,2’:6’,4”-terpyridine (4,2’:6’,4”-tpy) and 3,2’:6’,3”-terpyridine ligands is now a mature field, with the former ligand in particular being increasingly employed as a building block for the assembly of coordination polymers and networks [1,2]. There are a few examples of 4,2’:6’,4”-tpy ligands acting in a monodentate mode [3,4,5,6,7] but, typically, 4,2’:6’,4”-tpy coordinates through the two outer nitrogen atoms, with the central nitrogen atom non-coordinated. Therefore, ligands based upon a 4,2’:6’,4”-tpy metal-binding domain present moderately rigid, well-defined V-shaped motifs (Scheme 1a) which are ideal linkers in coordination assemblies. 

**Scheme 2:**
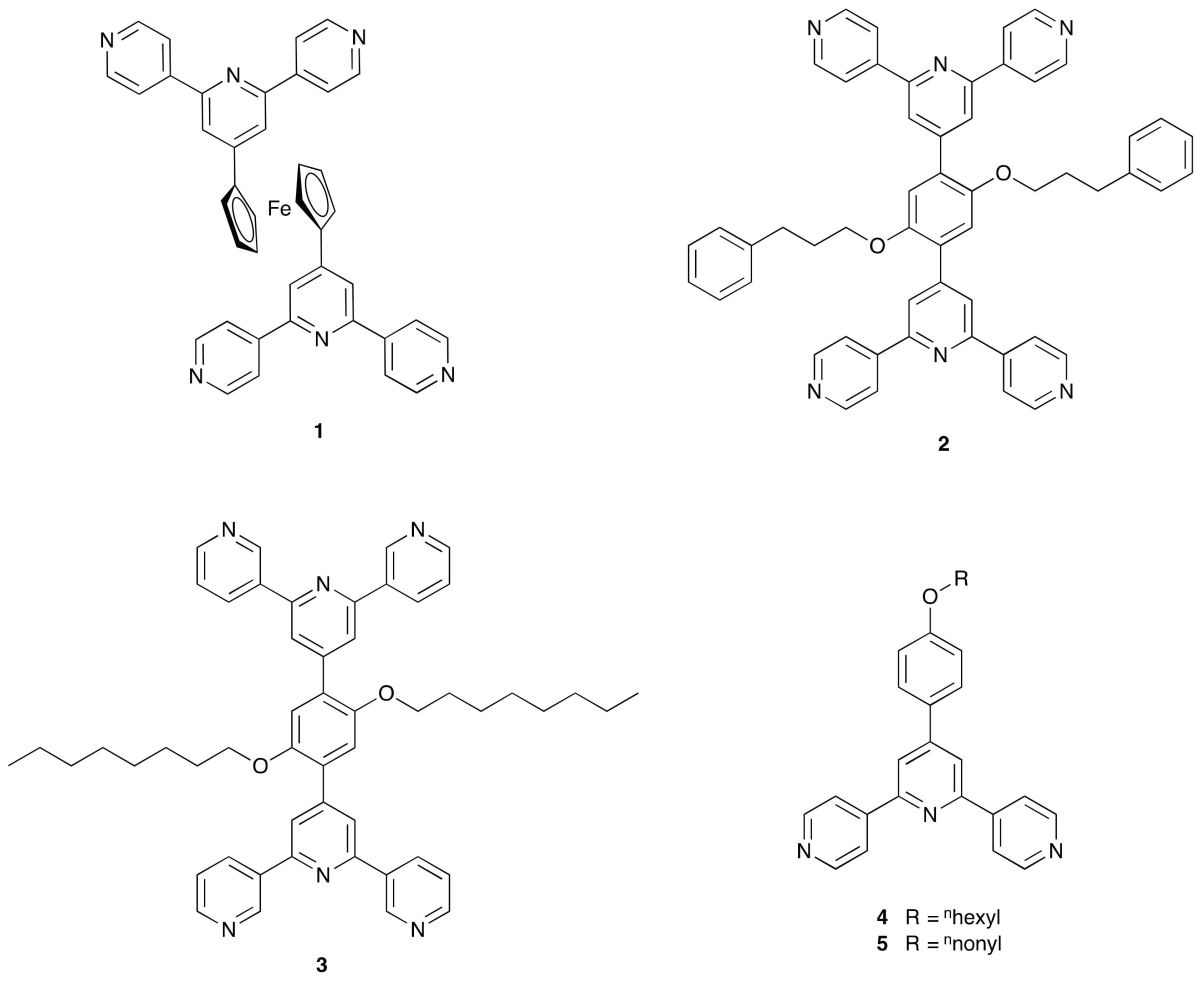
Structures of ditopic ligands **1** and **2** with 4,2’:6’,4”-tpy domains, and **3** with 3,2’:6’,3”-tpy metal-binding units and of the monotopic ligands **4** and **5**.

Starting with the 4,2’:6’,4”-tpy unit as a well-defined V-shaped building block, there are a number of strategies that can be applied to direct an assembly towards 2D- or 3D-architectures in preference to a 1D-chain. Since 4,2’:6’,4”-tpy is easily modified in the 4’-position using Kröhnke [8] or Hanan [9] syntheses, one approach is the incorporation of a coordinatively non-innocent domain D (Scheme 1b), for example D = pyridyl or carboxylate groups. The use of multitopic ligands [10,11,12] is an attractive way forward but has, so far, been little exploited. Scheme 1c illustrates a generic bis(4,2’:6’,4”-tpy) acting as a 4-connecting node. Rotation about the C–spacer bonds permits the node to have limiting planar or approximately tetrahedral geometries, and the directionality of the assembly can be modified by switching from 4,2’:6’,4”-tpy to 3,2’:6’,3”-tpy domains. We have recently employed such ditopic ligands in which the spacer (Scheme 1c) is a 1,4-C_6_H_4_ ring to assemble parallel interpenetrating 2D→2D sheets [13,14,15] and 3D-metal-organic frameworks (MOFs) [15,16]. Going to a ferrocen-1,1’-yl spacer introduces rotational freedom and, in [{Zn_2_(**1**)Cl_4_}∙3CHCl_3_]*_n_* where **1** = 1,1’-bis(4,2’:6’,4”-terpyridin-4’-yl)ferrocene (Scheme 2), the ligand adopts a cisoid-conformation, leading to a double-stranded 1D-polymer chain [17]. 

The discussion above focuses on bis(4,2’:6’,4”-tpy) or bis(3,2’:6’,3”-tpy) as a 4-connecting node. However, the choice of metal is naturally important since its preferred coordination number and geometry dictate whether it will act as a linker or node. When connected through ZnX_2_ units (X = halide) as in [Zn_2_Br_4_(**2**)∙H_2_O]*_n_* (**2** is shown in Scheme 2), ligand **2** acts as a 4-connecting node and the zinc(II) atoms are linkers; the assembly is a MOF consisting of 2-fold interpenetrating **nbo** nets [15]. In contrast, when ligand **3** (Scheme 2) is combined with Co(NCS)_2_, both the metal and ligand function as 4-connecting nodes (planar and approximately tetrahedral, respectively) and assemble into a 3D {4^2^.8^4^} **lvt** net [16]. Limiting the ligand to a single 4,2’:6’,4”-tpy domain as in **4** and **5** (Scheme 2) and reacting with Co(NCS)_2_ leads to the formation of 3D chiral **neb** nets [18]. This assembly is noteworthy in that a chiral MOF is built from achiral node and linkers. The presence of the long alkyl-tails appears to be important in directing or stabilizing these **neb** nets, since related 4,2’:6’,4”-tpy ligands bearing in the 4’-position ^t^Bu, Ph, 4-MeOC_6_H_4_, 4-EtOC_6_H_4_, 4-^n^PrOC_6_H_4_, 4-HC≡CC_6_H_4_, 1*H*-imidazol-4-yl or benzo[*d*][1,3]dioxol-5-yl substituents form 2D-nets with Co(NCS)_2_ [6,19,20,21,22]. With the exception of the work of Mondal et al. [20], crystal growth of the coordination networks described above was by layering under room temperature conditions. 

**Scheme 3:**
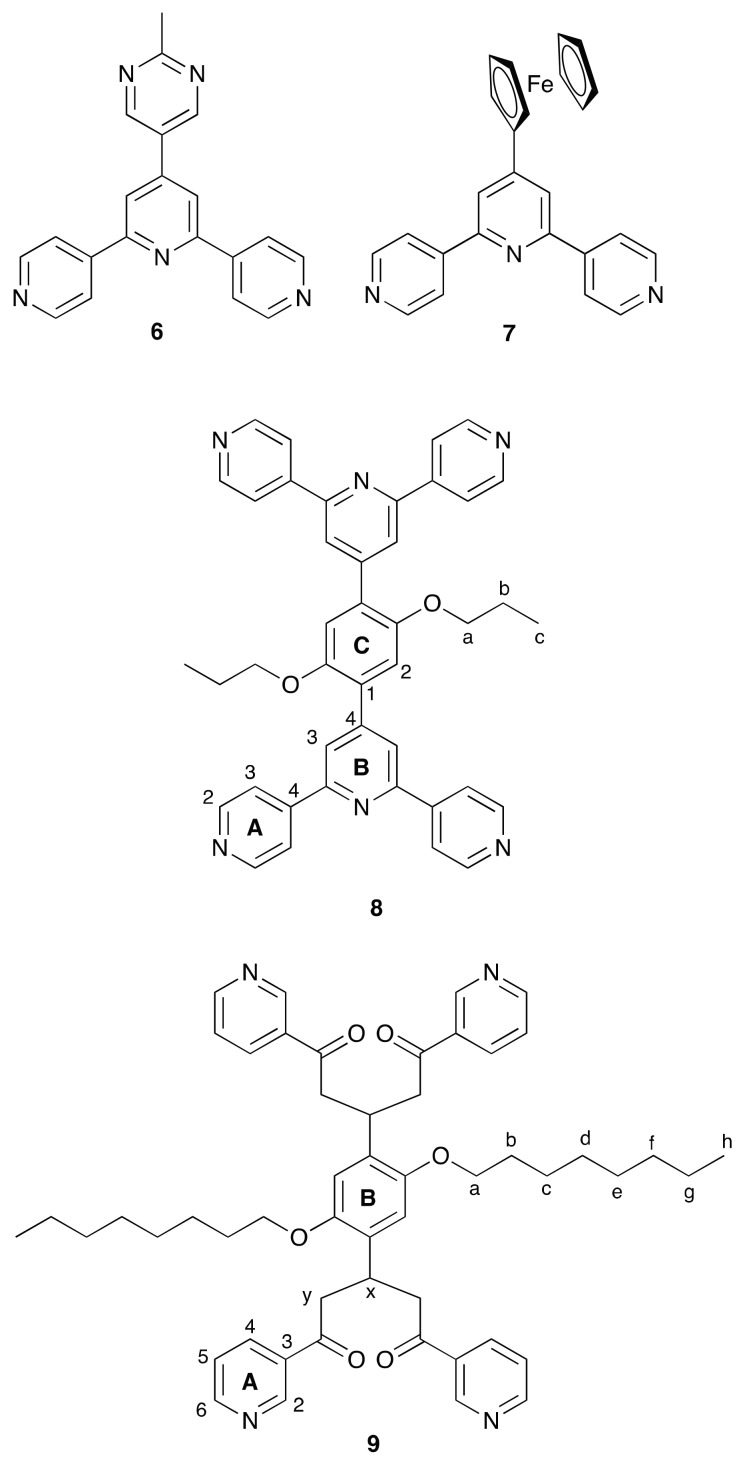
Structures of ligands **6**–**9** with atom labelling for NMR spectroscopic assignments in **8** and **9**.

We now present a comparative investigation of the coordination assemblies formed between Co(NCS)_2_ and two monotopic 4,2’:6’,4”-tpy ligands and two ditopic ligands. Ligand **6** (Scheme 3) contains a 2-methylpyrimidin-5-yl substituent and we were interested to see if this would be an additional metal-binding domain to cobalt(II). We have previously reported that the pyrimidinyl units in 4’-(pyrimidin-5-yl)-4,2’:6’,4”-terpyridine or **6** remain uncoordinated in reactions with zinc(II) halides [23], whereas Grafino et al. observe examples of both coordinated and non-coordinated pyrimidinyl *N*-donors in reactions of 4’-(4-(pyrimidin-5-yl)phenyl)- 4,2’:6’,4”-terpyridine with Zn(acac)_2_ [5]. Ligand **7** (Scheme 3) is related to ditopic ligand **1** (Scheme 2) but offers only one 4,2’:6’,4”-tpy domain. Both 1D-polymer chains and metallomacrocycles have been reported for combinations of **7** with ZnX_2_ (X = Cl, Br, I, SCN or OAc) [24,25]. The assemblies are competitive for ZnI_2_ and both the 1D-chain [{ZnI_2_(**7**)}∙2CHCl_3_]*_n_* [25] and discrete metallosquare [Zn_4_I_8_(**7**)_4_∙1.4MeOH] [24] have been crystallographically characterized. A feature that the ferrocenyl unit in **1** or **7** brings to crystal packing is its possibility for face-to-face π-stacking with pyridine rings [24]. Ditopic ligand **8** (Scheme 3) is a member of the 1,4-bis(alkoxy)-2,5-bis(4,2’:6’,4”-terpyridin-4’-yl)benzene family to which **2** (Scheme 2) belongs; while we have investigated assemblies formed between such ligands with zinc(II) halides [15], we have not described reactions with Co(NCS)_2_. Finally, ligand **9** (Scheme 3) is analogous to **3** in terms of the four *N*-donors. The flexibility of **9** is greater than that of **3**, and we wished to investigate how this would redirect the coordination assembly as compared to the 3D {4^2^.8^4^} **lvt** net in [Co(NCS)_2_(**3**)∙4CHCl_3_]*_n_* [16]. 

## 2. Results and Discussion

### 2.1. Ligand Syntheses and Characterization

**Scheme 4:**
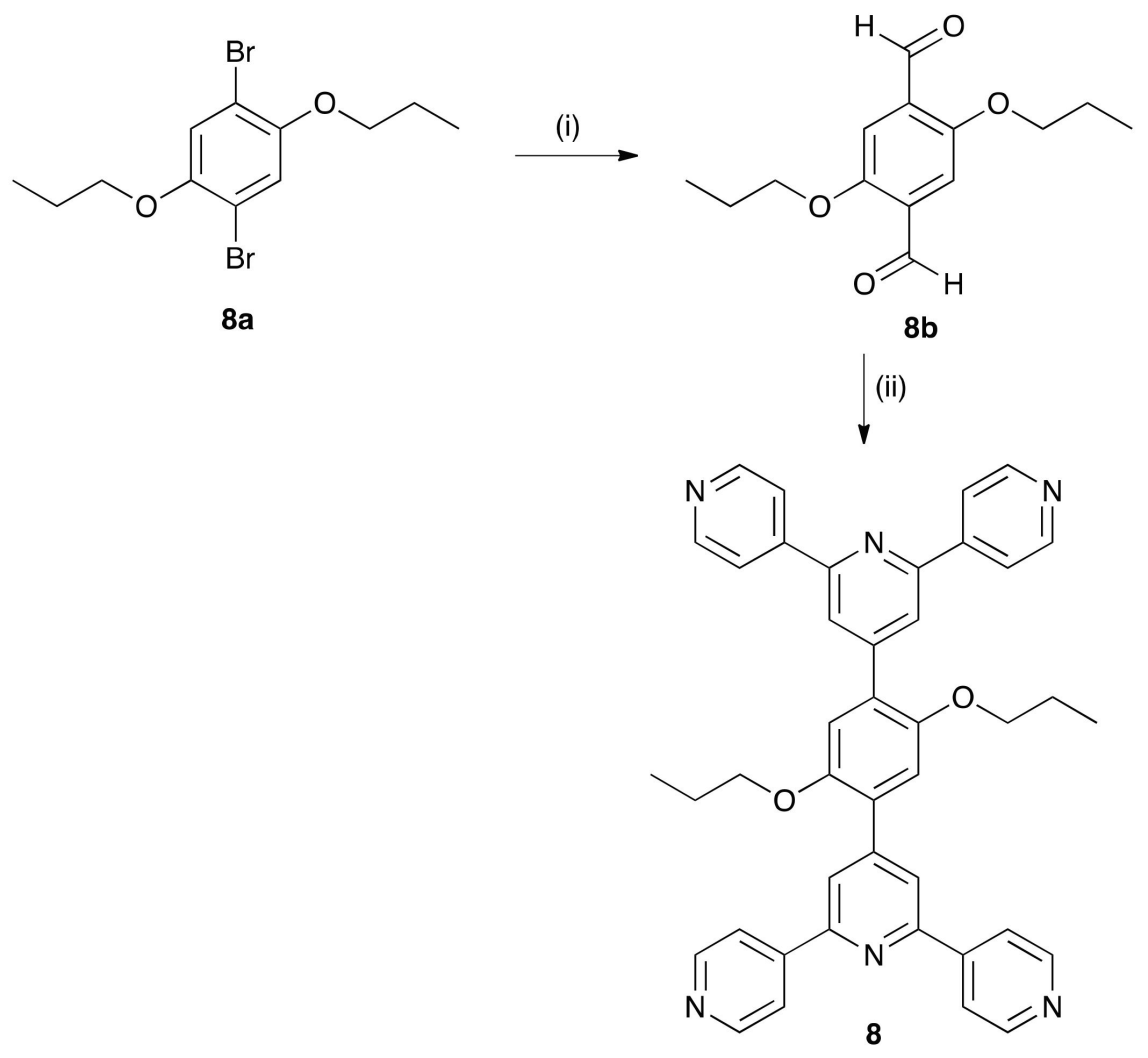
Synthetic route to compound **8**. Conditions: (**i**) ^n^BuLi, Et_2_O, 0 °C, 6 h; DMF, room temperature, yield 56.3%; and (**ii**) 4-acetylpyridine, KOH, EtOH, aqueous NH_3_, room temperature, yield 21.6%.

**Figure 1:**
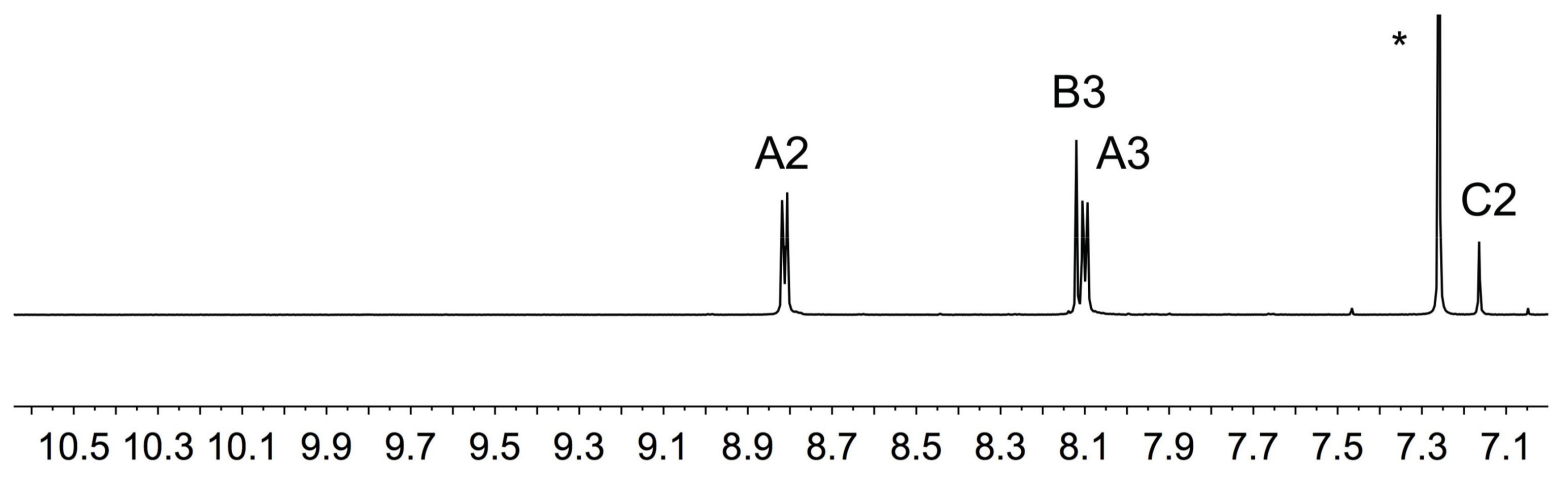
Part of the 500 MHz ^1^H NMR spectrum of **8** in CDCl_3_ showing the aromatic region of **8** and confirming the loss of both aldehyde groups of the precursor **8b**. * = residual CHCl_3_.

Ligands **6** and **7** were prepared as previously reported [23,25]. The synthetic route to **8** is summarized in Scheme 3 and is closely related to the synthesis of **2** [15]. Compound **8a** (Scheme 3) was prepared from the commercially available 2,5-dibromohydroquinone and then reacted with ^n^BuLi followed by DMF to yield the dialdehyde **8b**. The ^1^H NMR spectrum of **8b** was consistent with previously reported data [26]. The one-pot method of Hanan [9] (Scheme 3) was then used to transform **8b** into compound **8**. The disappearance of the aldehyde signal at δ 10.52 ppm confirmed that derivatization of both aldehyde groups had occurred (Figure 1). The ^1^H and ^13^C NMR spectra of compound **8** were assigned using COSY, HMQC, HMBC and NOESY methods and were in accord with the structure shown in Scheme 4.

**Scheme 5:**
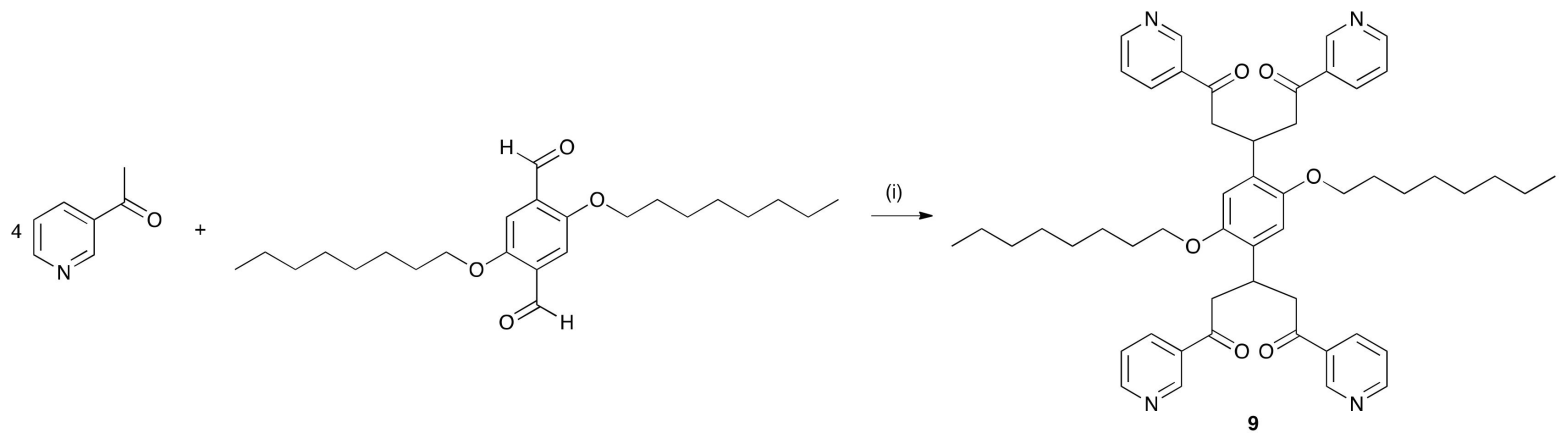
Synthetic route to compound **9**. Conditions: (**i**) KOH, EtOH, room temperature, yield 31.2%.

**Figure 2:**
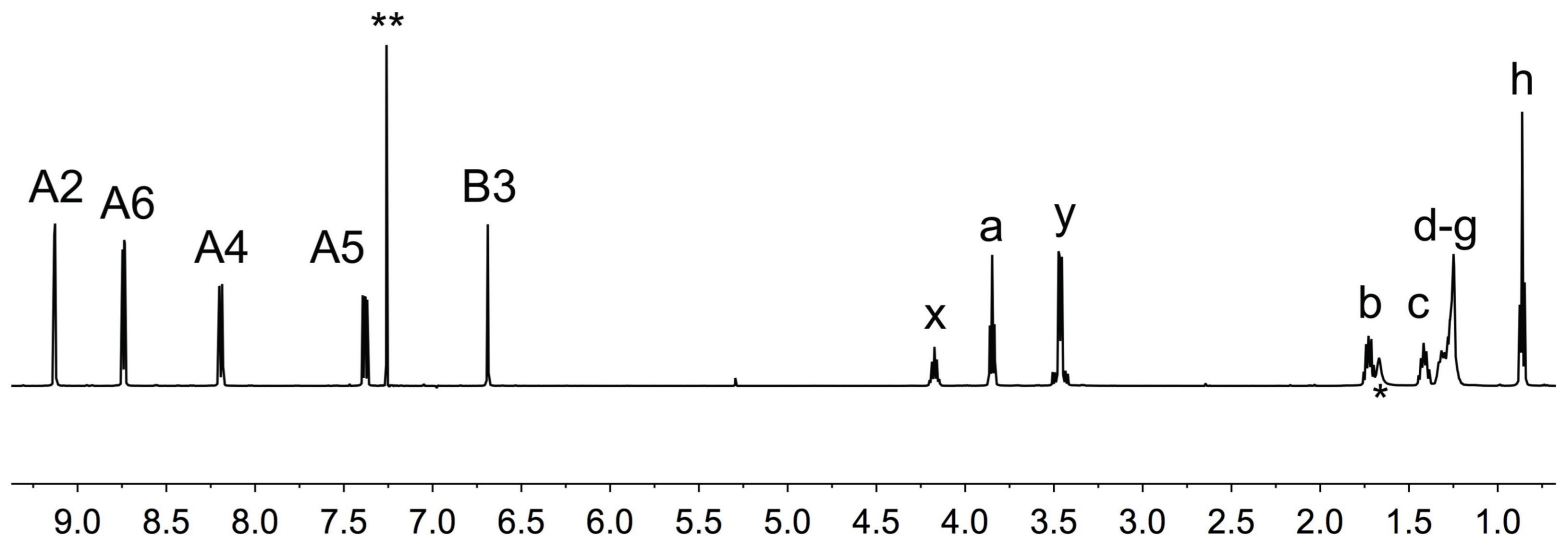
The 500 MHz ^1^H NMR spectrum of **9** in CDCl_3_. ** = residual CHCl_3_; * = water. See Scheme 3 for atom labelling.

Compound **9** was prepared by the reaction of four equivalents of 3-acetylpyridine with 2,5-bis(octoxy)benzene-1,4-dicarbaldehyde under basic conditions (Scheme 5). In the electrospray mass spectrum of **9**, the base peak at *m*/*z* 839.4 corresponded to [M+H]^+^. Figure 2 shows the ^1^H NMR spectrum of **9**. Both this and the ^13^C NMR spectrum were assigned using 2D methods (Figure S1 shows the HMQC spectrum) and were consistent with the structure shown in Scheme 5. In the ^13^C NMR spectrum, a resonance at *δ* 198.2 ppm characterized the carbonyl groups and a band at 1681 cm^−1^ arising from the C=O stretch was observed in the IR spectrum of **9**.

### 2.2. 4’-(2-Methylpyrimidin-5-yl)-Functionalization: Assembly of a 1D-Chain

**Figure 3:**
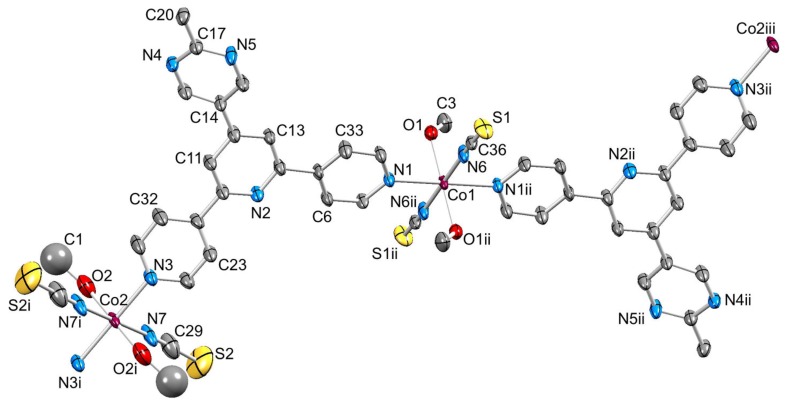
Structure of the repeat unit in {[Co_2_(NCS)_4_(MeOH)_4_(**6**)_2_]∙2MeOH∙8H_2_O}*_n_* with symmetry generated atoms; H atoms and solvent molecules are omitted. Ellipsoids are plotted at 40% probability; atom C1 was refined isotropically (see text). Symmetry codes: i = 1 − *x*, 2 − *y*, 1 − *z*; ii = 1 − *x*, −*y*, 2 − *z*; iii = *x*, −2 + *y*, 1 + *z*. Selected bond parameters: Co1–N1 = 2.189(6), Co1–N6 = 2.095(7), Co1–O1 = 2.098(6), Co2–N3 = 2.154(6), Co2–N7 = 2.069(7), Co2–O2 = 2.077(9) Å; N1–Co1–N6 = 88.8(2), N1–Co1–O1 = 86.5(2), N3–Co2–N7 = 90.1(2), N3–Co2–O2 = 91.8(3), N7–Co2–O2 = 90.9(4)°.

**Figure 4:**
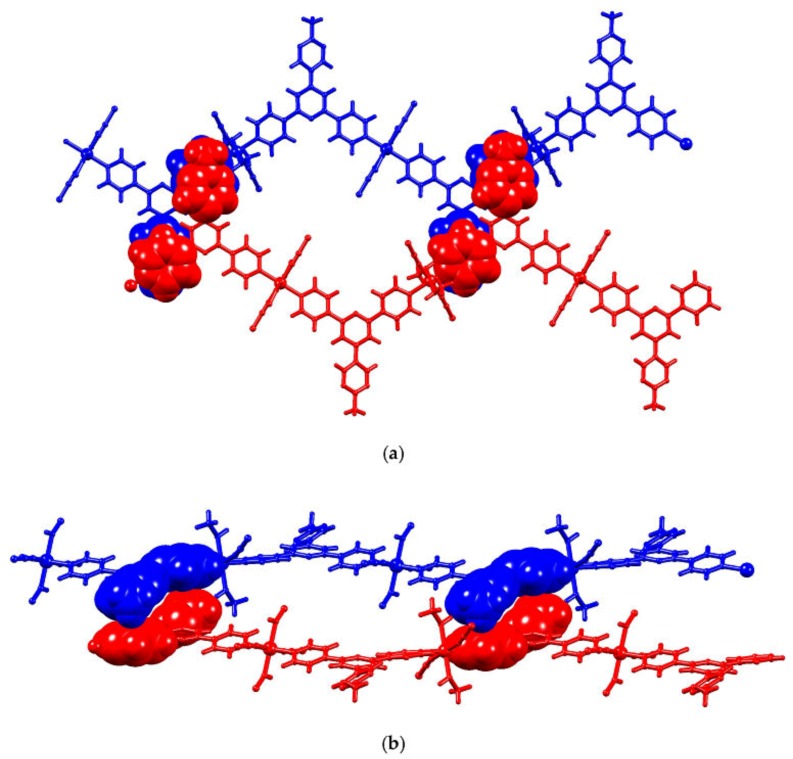
Parts of two adjacent 1D chains in {[Co_2_(NCS)_4_(MeOH)_4_(**6**)_2_]∙2MeOH∙8H_2_O}*_n_*. (**a**) View showing the zigzag profile of the chain and association between pyridine and pyrimidine rings in adjacent chains; (**b**) The same polymer units as in (**a**) viewed down the crystallographic *a*-axis.

Layering of a methanol solution of Co(NCS)_2_ over a chloroform solution of **6** yielded a few X-ray quality pink crystals under ambient conditions over a period of 2–4 weeks. Single-crystal X-ray diffraction confirmed a formulation of {[Co_2_(NCS)_4_(MeOH)_4_(**6**)_2_]∙2MeOH∙8H_2_O}*_n_*, but there was insufficient material for characterization of the bulk sample by powder diffraction. The compound crystallizes in the triclinic *P*–1 space group, and Figure 3 shows the structure of the repeat unit in the coordination polymer. Selected bond distances and angles are given in the figure caption. Ligand **6** binds to cobalt through atoms N1 and N3 of the 4,2’:6’,4”-tpy, leaving the central pyridine N-donor and the two pyrimidinyl N-donors uncoordinated. The two crystallographically independent cobalt atoms are in similar octahedral coordination spheres, and since each of Co1 and Co2 resides on an inversion centre, the pairs of 4,2’:6’,4”-tpy, thiocyanato and MeOH ligands are necessarily *trans* to one another (Figure 3). The MeOH molecule containing C1 is disordered (50:50 site occupancies), each site sharing a common O2 atom. Since each of Co1 and Co2 binds two ligands **6** which are mutually *trans*, each metal centre acts as a linear, 2-connecting node. The structure in Figure 3 propagates into a 1D-zigzag chain and as shown in Figure 4a. Coordination by the MeOH solvent blocks two cobalt coordination sites precluding propagation into a higher-dimensional network. The zigzag chains lie over one another such that one pyridine ring of the 4,2’:6’,4”-tpy unit stacks over a pyrimidine ring in the next chain. However, the stacking is not optimal. Although the pyridine_centroid_...pyrimidine_plane_ separation is 3.35 Å, the angle between the planes of the ring containing N1 and pyrimidine ring containing N4^iv^/N5^iv^ (symmetry code iv = − *x*, 1 − *y*, 2 − *z*) is 23.8° (Figure 4b). The presence of the methyl substituent on the pyrimidine ring may sterically hinder a more optimal π-stacking arrangement. 

**Figure 5:**
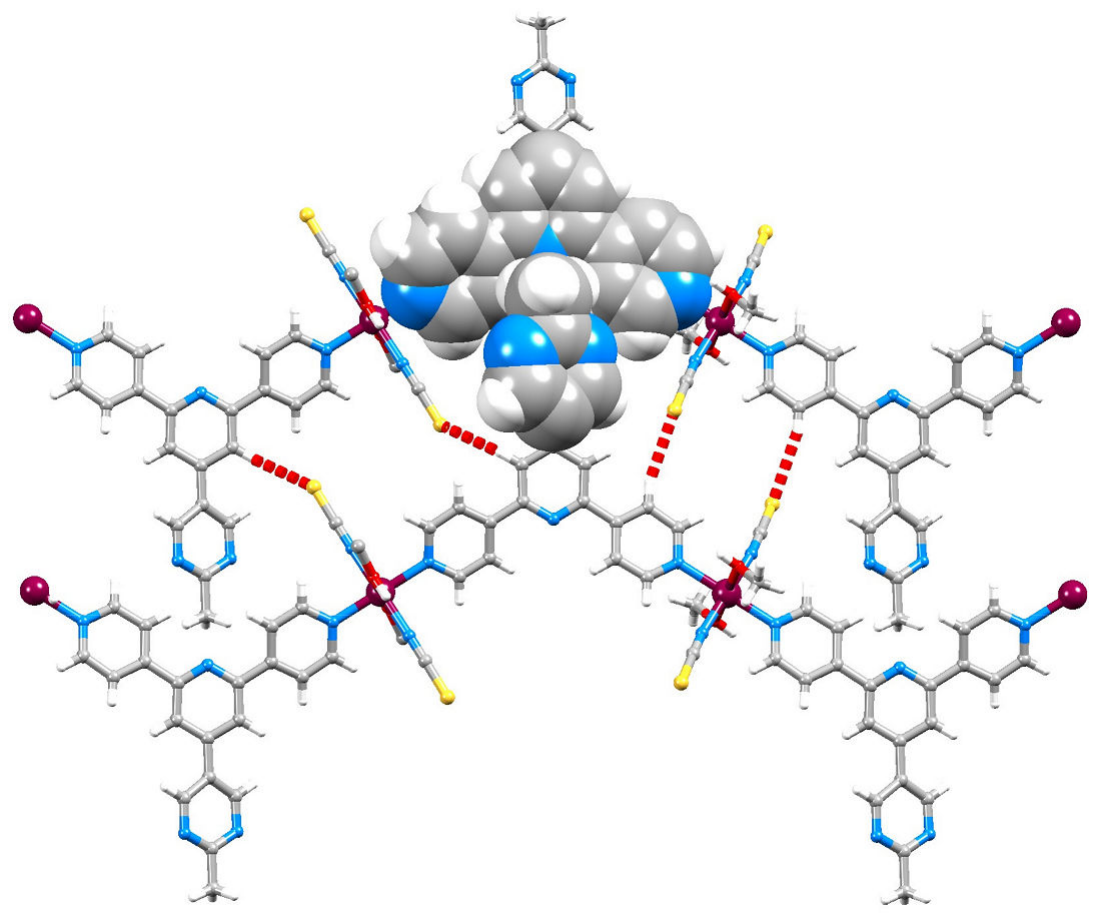
Arrangement of zigzag chains in {[Co_2_(NCS)_4_(MeOH)_4_(**6**)_2_]∙2MeOH∙8H_2_O}*_n_* showing short S...H contacts (hashed red lines).

Ligands based on 4,2’:6’,4”-tpy feature in a large number of 1D zigzag chains in which the metal nodes are 2-connecting {M_2_(μ-OAc)_4_} paddle-wheel units [1]. A common structural motif in these assemblies is the accommodation of the 4’-substituent of the 4,2’:6’,4”-tpy within the V-shaped cleft of a 4,2’:6’,4”-tpy domain in an adjacent chain leading to planar 2D-sheets with a herringbone packing motif. In {[Co_2_(NCS)_4_(MeOH)_4_(**6**)_2_]∙2MeOH∙8H_2_O}*_n_*, rather than being accommodated within the V-shaped pocket of the adjacent chain, the methyl group of the 4’-(2-methylpyrimidin-5-yl) substituent sits over the central N2 atom of the chain beneath it (C_Me_...N = 3.42(1) Å) as shown in Figure 5. This arrangement of neighbouring chains leads to short S...H contacts [27] as shown in Figure 5. The S...H distances of 2.96 and 2.82 Å (the H atoms are in calculated positions) compare with 3.00 Å for the sum of the H and S van der Waals radii using Bondi values [28], although this value may be an overestimate [29,30]. An analysis by Rowland and Taylor [30] of crystallographic data for intermolecular contacts in organic compounds suggests that the Bondi van der Waals radius for H of 1.2 Å is overestimated by 0.1 Å. 

Cavities in the lattice in {[Co_2_(NCS)_4_(MeOH)_4_(**6**)_2_]∙2MeOH∙8H_2_O}*_n_* are occupied by H_2_O and MeOH molecules. There are (in the asymmetric unit) two independent MeOH molecules, each half-occupancy. The lattice water molecules comprise three independent full-occupancy molecules and two independent half-occupancy molecules. Because of the fractional occupancies, discussion of any hydrogen-bonded interactions involving solvent molecules is not warranted.

### 2.3. Ferrocenyl-Functionalization: Assembly of a 2D-Network

**Figure 6:**
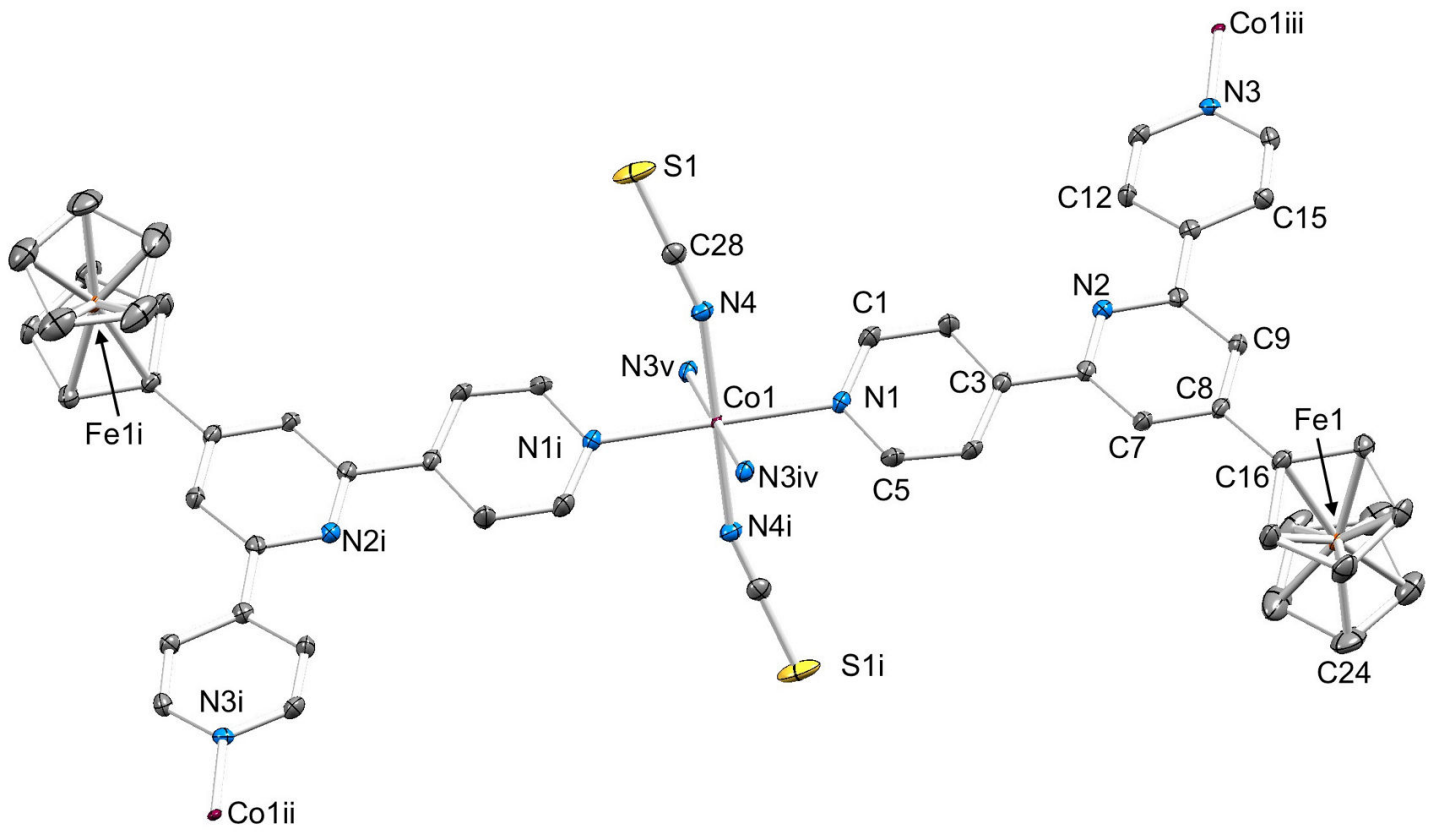
Structure of the repeat unit in {[Co(NCS)_2_(**7**)_2_]∙4CHCl_3_}*_n_* with symmetry generated atoms; H atoms and solvent molecules are omitted. Ellipsoids are plotted at 50% probability. Symmetry codes: i = −*x*, 1 − *y*, 1 − *z*; ii = −^1^/_2_ + *x*, ^3^/_2_ − *y*, −^1^/_2_ + *z*; iii = ^1^/_2_ + *x*, ^1^/_2_ − *y*, ^1^/_2_ + *z*; iv = −^1^/_2_ + *x*, ^1^/_2_ − *y*, −^1^/_2_ + *z*; v = ^1^/_2_ − *x*, ^1^/_2_ + *y*, ^3^/_2_ + *z*. Important bond parameters: Co1–N1 = 2.183(2), Co1–N3^iv^ = 2.183(2), Co1–N4 = 2.083(2), C8–C16 = 1.472(3) Å; N–Co1–N angles are in the range 86.77(8)–93.23(8)°.

**Figure 7:**
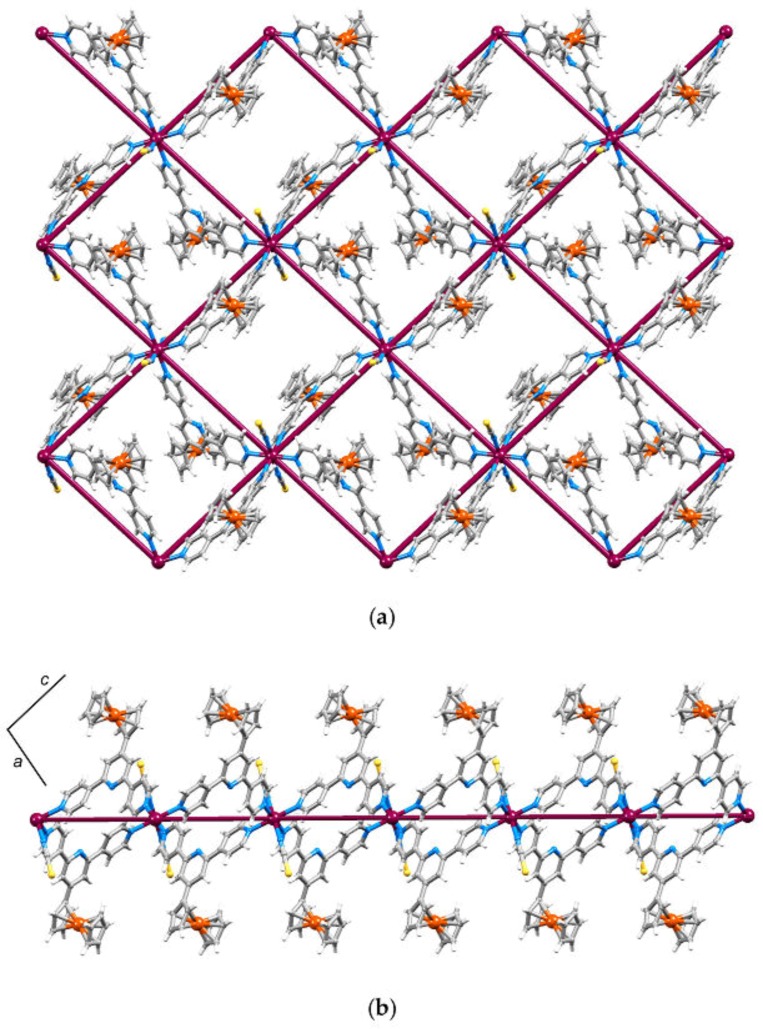
(**a**) TOPOS [31] representation of part of one (4,4) sheet in {[Co(NCS)_2_(**7**)_2_]∙4CHCl_3_}*_n_* overlaid with the structure; and (**b**) the same part of the sheet viewed down the crystallographic *b*-axis.

**Figure 8:**
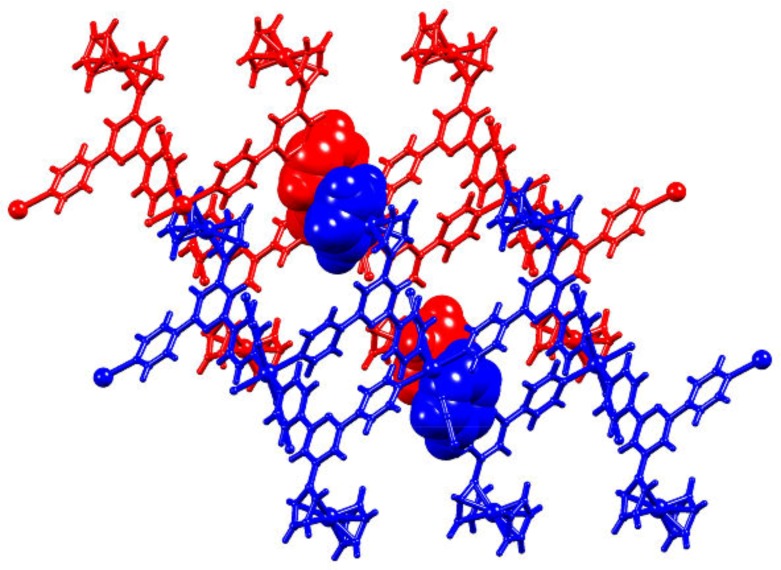
Parts of two adjacent sheets in {[Co(NCS)_2_(**7**)_2_]∙4CHCl_3_}*_n_* showing the π-stacking between a ferrocenyl ring in one sheet and pyridyl ring in the next sheet.

Layering of a methanol solution of Co(NCS)_2_ over a chloroform solution of **7** resulted in the growth of orange crystals of {[Co(NCS)_2_(**7**)_2_]∙4CHCl_3_}*_n_*. An X-ray quality crystal was chosen for single-crystal structure determination and the bulk sample was characterized by powder diffraction (Figure S2). The compound crystallizes in the monoclinic *P*2_1_/*n* space group. Figure 6 depicts the octahedral coordination environment of the cobalt(II) centre and selected bond parameters are given in the figure caption. Atom Co1 lies on an inversion centre and is bound by two thiocyanato ligands (necessarily *trans*) and four ligands **7**. The near square-planar arrangement of N1, N1i, N3v and N3iv (Figure 6) leads to the metal being a planar, 4-connecting node. The structure propagates into a (4,4) net (Figure 7) and belongs to the family of (4,4) nets which have been reported for combinations of Co(NCS)_2_ and a number of 4’-functionalized 4,2’:6’,4”-tpy ligands (functionality = ^t^Bu, Ph, 4-MeOC_6_H_4_, 4-EtOC_6_H_4_, 4-^n^PrOC_6_H_4_, 4-HC≡CC_6_H_4_, 1*H*-imidazol-4-yl or benzo[*d*][1,3]dioxol-5-yl [6,19,20,21,22]. As Figure 7b shows, the ferrocenyl units protrude above and below the 2D-sheet, and adjacent sheets are interlocked with each ferrocenyl unit of one sheet lying over a 4,2’:6’,4”-tpy unit in the next sheet. The 4,2’:6’,4”-tpy unit deviates slightly from planarity with angles between the planes of the pyridine rings containing N1/N2 and N2/N3 of 11.4 and 13.5°, respectively. The cyclopentadienyl ring containing C16 is twisted 16.0° with respect to the pyridine ring with N2. We have previously described the role of face-to-face π-stacking between ferrocenyl and pyridyl units in crystal packing [24]. Similar effects are observed in {[Co(NCS)_2_(**7**)_2_]∙4CHCl_3_}*_n_* and Figure 8 illustrates that adjacent (4,4) sheets associate through stacking interactions. These involve the pyridine ring containing N1 and cyclopentadienyl ring containing C24^v^ (symmetry code v = −1 + *x*, *y*, *z*). The angle between the ring planes is 6.9° and the pyridine_centroid_...C_5_-ring_plane_ distance is 3.22 Å; the distance of 4.17 Å between the ring centroids is rather long for an optimal interaction. The pyridine ring containing N3 also lies over a ferrocenyl unit (that with C16^vi^, symmetry code vi = 1 − *x*, −*y*, 1−*z*), but, in this case, the inter-plane angle is 19.2°, leading to an inefficient π-stacking contact.

### 2.4. To a 3D-Network: {[Co(NCS)_2_(8)_2_]^.^2C_6_H_4_Cl_2_}_n_

**Figure 9:**
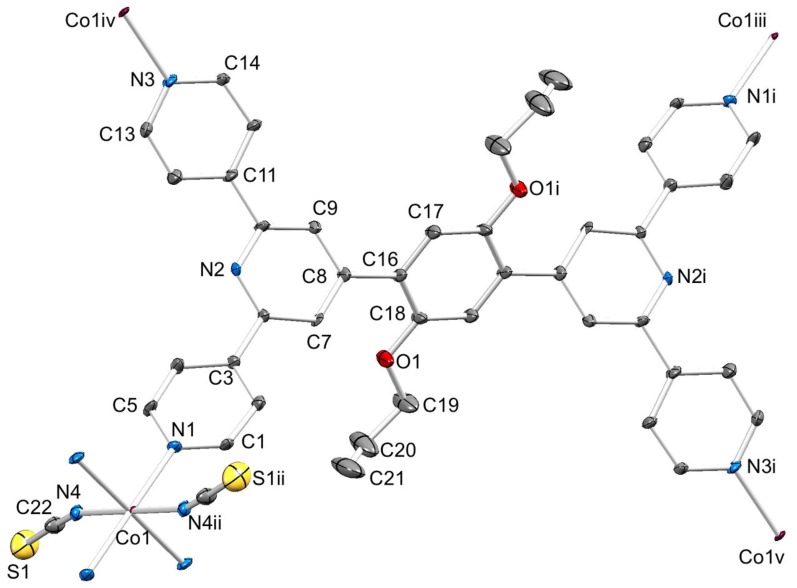
Structure of the repeat unit in {[Co(NCS)_2_(**8**)_2_]∙2C_6_H_4_Cl_2_}*_n_* with symmetry generated atoms; H atoms and solvent molecules are omitted. Ellipsoids are plotted at 40% probability. Symmetry codes: i = −*x*, 2 − *y*, 1 − *z*; ii = 1 − *x*, 1 − *y*, 1 − *z*; iii = −1 + *x*, 1 + *y*, *z*; iv = *x*, ^3^/_2_ − *y*, −^1^/_2_ + *z*; v = −1 + *x*, ^3^/_2_ + *y*, ^1^/_2_ + *z*. Selected bond parameters: Co1–N1 = 2.173(2), Co1–N4 = 2.047(3), Co1^iv^–N3 = 2.193(2), O1–C18 = 1.361(4), O1–C19 = 1.431(4) Å; N1–Co1–N4 = 90.47(10)°.

**Figure 10:**
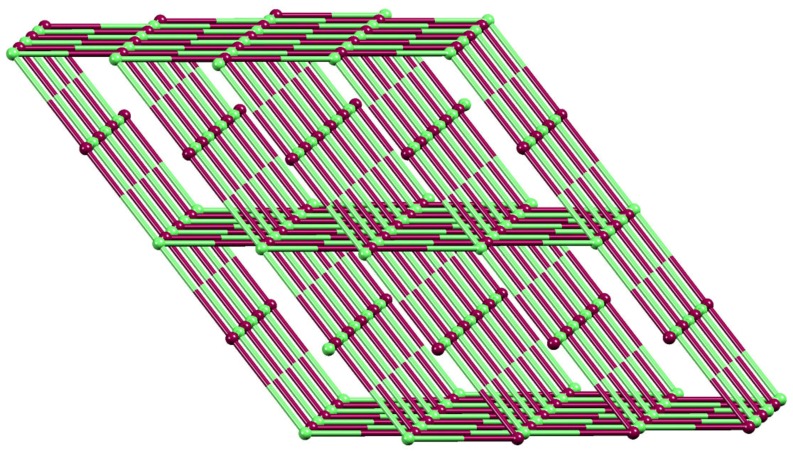
TOPOS [31] representation of part of the **cds** net in {[Co(NCS)_2_(**8**)_2_]∙2C_6_H_4_Cl_2_}*_n_*. Both Co (maroon) and ligand (green) are planar, 4-connecting nodes.

**Figure 11:**
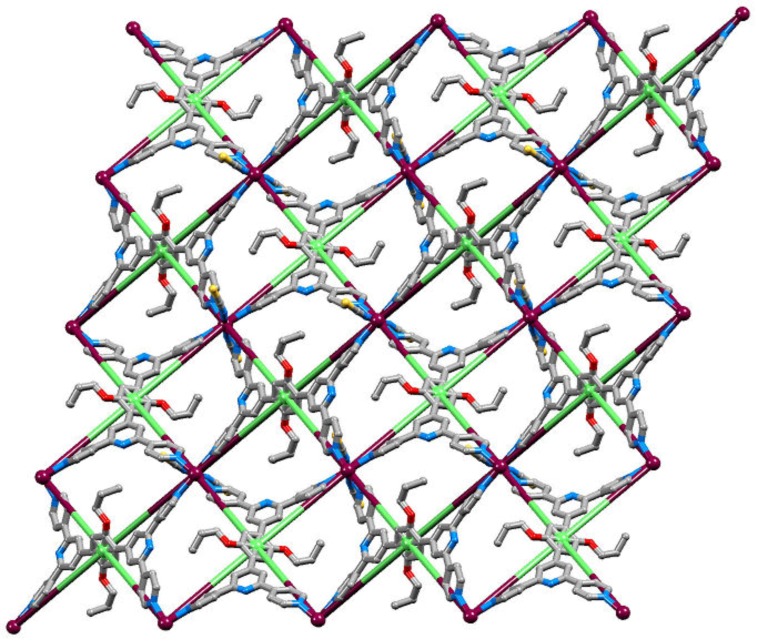
Overlay of the structure of {[Co(NCS)_2_(**8**)_2_]∙2C_6_H_4_Cl_2_}*_n_* (H atoms and solvent molecules omitted) on a TOPOS [31] representation of part of the **cds** net. The ligand node (green) is defined as the centroid of the arene ring.

Ligand **8** presents two 4,2’:6’,4”-tpy units connected in a “back-to-back” fashion through a spacer which contains two *n-*propoxy tails. We have demonstrated that the length of such tails can have a significant impact on the outcome of assembly processes [13,14,15]. Single crystals grown by layering a MeOH solution of Co(NCS)_2_ over a CHCl_3_ solution of **8** were not of X-ray quality. However, a change to a 1,2-dichlorobenzene solution of **8** led to crystals that were harvested after a few weeks, and single crystal X-ray diffraction showed the formation of a coordination network of formula {[Co(NCS)_2_(**8**)_2_]∙2C_6_H_4_Cl_2_}*_n_*. The compound crystallizes in the monoclinic space group *P*2_1_/*c* and the repeat unit in the structure is depicted in Figure 9. Atom Co1 is octahedrally sited and since it resides on an inversion centre, the two thiocyanato ligands are necessarily *trans*. Atom Co1 binds to four different ligands **8** (Figure 9) and thereby functions as a planar, 4-connecting node. Selected bond parameters within the coordination sphere are given in the caption to Figure 9, and the N_tpy_–Co–N_tpy_ bond angles that define the planar node are in the range 84.44(8)–95.56(8)°. The asymmetric unit contains half of a molecule of **8**, and the second half is generated by inversion (Figure 9). Thus, like the cobalt centre, the ditopic ligand also acts as a planar 4-connecting node. The 4,2’:6’,4”-tpy unit deviates from planarity with angles between the rings containing N1/N2 and N2/N3 being 31.3 and 19.5°, respectively. The twist of the pyridine ring with N2 with respect to the central aryl ring is 40.5°, thereby minimizing inter-ring repulsive H...H contacts. The combination of 4-connecting metal and ligand nodes leads to the assembly of a 3D {6^5^.8} **cds** [32,33] net, a TOPOS [31] representation of which is shown in Figure 10. In a **cds** net, half of the adjacent nodes are perpendicular to one another and half are coplanar [33]. The preference for the **cds** architecture is in contrast to the assembly of the {4^2^.8^4^} **lvt** net which forms from Co(NCS)_2_ and ditopic ligand **3** (Scheme 2) [16]. Ligands **3** and **8** both act as 4-connecting nodes, but differ in having two 3,2’:6’,3”-tpy or 4,2’:6’,4”-tpy domains, respectively. In addition to a change in the vectorial properties of the ligands, ligand **8** possesses shorter alkoxy tails than **3** (*n*-propoxy versus *n*-octoxy). Figure 11 shows an overlay of the structure on the TOPOS representation of the net and this orientation reveals the accommodation of the extended propoxy tails within cavities in the network. Remaining cavities in the **cds** net are occupied by 1,2-dichlorobenzene molecules (see Experimental Section). The bulk sample was characterized by powder diffraction (Figure S3). 

### 2.5. Relaxing the Backbone: Going from an **lvt** Net to a 2D-Sheet

**Figure 12:**
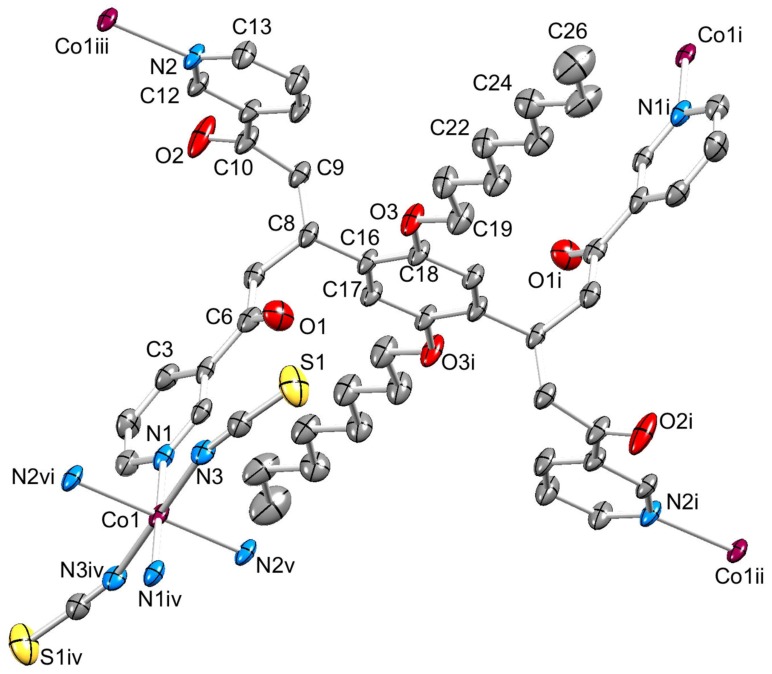
Structure of the repeat unit in {[Co(NCS)_2_(**9**)]∙2CHCl_3_}*_n_* with symmetry generated atoms; H atoms and solvent molecules are omitted. Ellipsoids are plotted at 40% probability. Symmetry codes: i = −1 + *x*, 1 + *y*, 1 + *z*; ii = −1 + *x*, *y*, 1 + *z*; iii = *x*, −1 + *y*, *z*; iv = 2 − *x*, 3 − *y*, −*z*; v = *x*, 1 + *y*, *z*; vi = 2 − *x*, 2 − *y*, −*z*. Selected bond parameters: Co1–N1 = 2.196(4), Co1–N3 = 2.070(4), Co1–N2^v^ = 2.199(4), O1–C6 = 1.216(6), O2–C10 = 1.193(7), O3–C18 = 1.383(6), O3–C19 = 1.413(6) Å; N1–Co1–N3 = 91.13(17), N_tpy_–Co1–N_tpy_ range 87.68(16)–92.32(16)°.

As described above, ditopic ligand **3** (Scheme 2) reacts with Co(NCS)_2_ under conditions of crystal growth by layering to give {[Co(NCS)_2_(**3**)]∙4CHCl_3_}*_n_* which possesses a {4^2^.8^4^} **lvt** net [16]. This assembly is rather uncommon among MOFs consisting of 4-connected nets [32] and in {[Co(NCS)_2_(**3**)]∙4CHCl_3_}*_n_*, the **lvt** net is produced by a combination of planar Co nodes and approximately tetrahedral ligand nodes. We decided to investigate the effects of modifying the ligand structure so as to retain a 4-connecting domain with similar metrics to **3** but with a more flexible backbone. Ligand **9** (Scheme 3) conforms to these criteria. A methanol solution of Co(NCS)_2_ was layered over a chloroform solution of **9** and, after 2–4 weeks, X-ray quality crystals had grown. Single-crystal X-ray diffraction analysis confirmed the formation of {[Co(NCS)_2_(**9**)]∙2CHCl_3_}*_n_* with a Co:ligand ratio equivalent to that in {[Co(NCS)_2_(**3**)]∙4CHCl_3_}n [16]; the bulk sample was characterized by powder diffraction (Figure S4). The compound crystallizes in the triclinic space group *P*–1 with atom Co1 lying on an inversion centre. The repeat unit is shown in Figure 12 and the coordination environment of Co1 resembles that in {[Co(NCS)_2_(**8**)_2_]∙2C_6_H_4_Cl_2_}*_n_* (Figure 9). Metrical parameters for the coordination sphere (caption to Figure 12) are unexceptional. The closeness of each N_tpy_–Co1–N_tpy_ angle to 90° leads to a square-planar metal node. The asymmetric unit contains half of a ligand **9**, and the second half is generated by inversion leading to **9** being a planar 4-connecting node (Figure 12), the centre of which is defined by the centroid of the arene ring containing atom C16. The planarity of the ligand **9** node in {[Co(NCS)_2_(**9**)]∙2CHCl_3_}*_n_* contrasts with the tetrahedral node that **3** presents in {[Co(NCS)_2_(**3**)]∙4CHCl_3_}*_n_* [16]; recall from the introduction that ditopic ligands of the type exemplified by **2** or **3** (Scheme 2) have limiting planar or approximately tetrahedral geometries. A comparison of the conformations of the coordinated ligands **3** and **9** in {[Co(NCS)_2_(**3**)]**∙**4CHCl_3_}*_n_* and {[Co(NCS)_2_(**9**)]**∙**2CHCl_3_}*_n_*, respectively, is shown in Figure S5.

**Figure 13:**
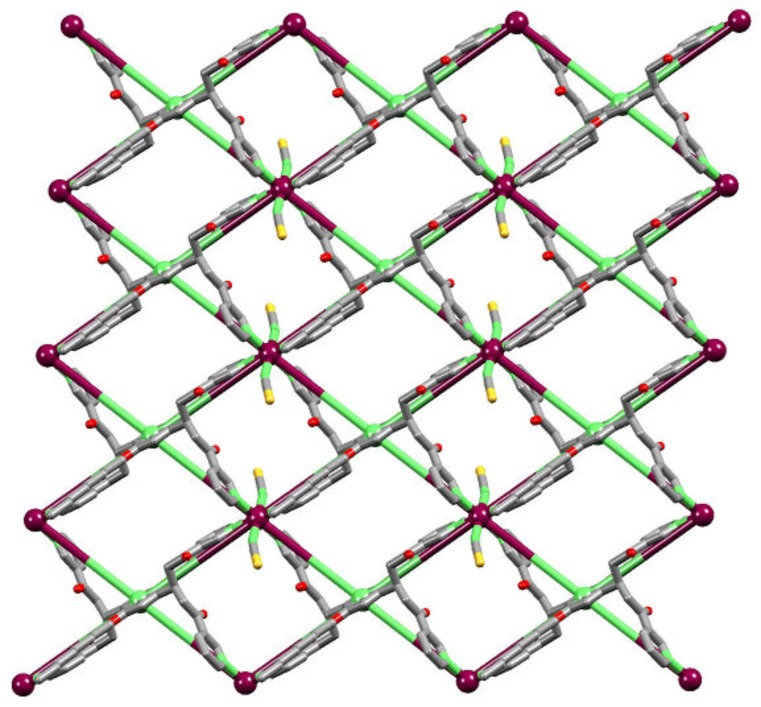
Overlay of the structure of {[Co(NCS)_2_(**9**)]∙2CHCl_3_}*_n_* (H atoms and solvent molecules omitted) on a TOPOS [31] representation of part of a 2D-sheet. The ligand node (green) is defined as the centroid of the central arene ring in **9**.

**Figure 14:**
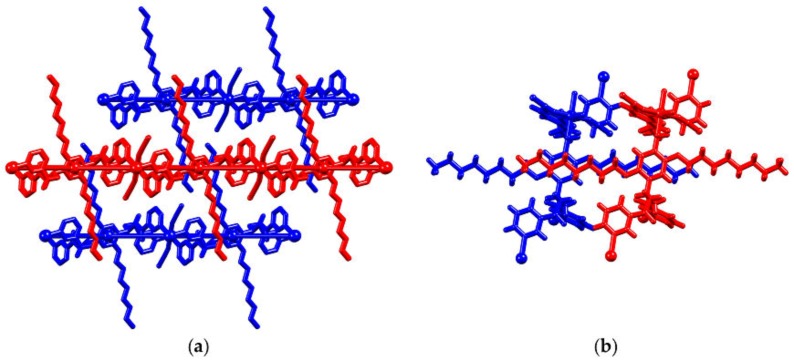
(**a**) Parts of three adjacent 2D-sheets in {[Co(NCS)_2_(**9**)]∙2CHCl_3_}*_n_* (H atoms and solvent molecules omitted) showing threading of the octoxy tails through neighbouring sheets; and (**b**) alignment of octoxy tails in adjacent sheets.

Propagation of the unit in Figure 12 leads to a 2D (4,4) net. In contrast to the (4,4) net in {[Co(NCS)_2_(**7**)_2_]∙4CHCl_3_}*_n_*, which is defined only by the metal nodes, the net in {[Co(NCS)_2_(**9**)]∙2CHCl_3_}*_n_* is defined by a combination of metal and ligand 4-connected nodes (Figure 13). The one crystallographically independent octoxy chain in {[Co(NCS)_2_(**9**)]∙2CHCl_3_}*_n_* is ordered and has a fully extended conformation. These features are associated with the threading of the octoxy tail through an adjacent sheet (Figure 14a) and the alignment of the tails (Figure 14b). Both van der Waals and C–H...π interactions play a role with inter-chain C...C and C...O separations of 4.35 and 4.33 Å and a close C–H...π contact of 3.26 Å (C...π distance = 4.04 Å) [34]. 

## 3. Materials and Methods

### 3.1. General

^1^H and ^13^C NMR spectra were recorded on a Bruker DRX-500 NMR spectrometer with chemical shifts referenced to residual solvent peaks (TMS = *δ* 0 ppm). Electrospray ionisation (ESI) mass spectra were measured on a Bruker esquire 3000plus spectrometer or Shimadzu LCMS-2020 instrument and high resolution ESI mass spectra on a Bruker maXis 4G QTOF instrument. The IR spectrum of **9** was recorded on a Perkin Elmer Spectrum Two (UATR) FT-IR.

3-Acetylpyridine, 4-acetylpyridine, 1-bromopropane, 2,5-dibromohydroquinone and 2,5-bis(octoxy)benzene-1,4-dicarbaldehyde were purchased from Sigma-Aldrich (Switzerland) and used without further purification. Ligands **6** and **7** were prepared as previously reported [23,25].

### 3.2. Synthesis of ***8a***

2,5-Dibromohydroquinone (2.0 g, 7.47 mmol), 1-bromopropane (1.72 mL, 2.32 g, 18.7 mmol) and anhydrous K_2_CO_3_ (3.1 g, 22.4 mmol) were added to dry DMF (100 mL) and the mixture was heated at 100 °C for 16 h. The mixture was cooled to room temperature then poured into a beaker containing ice water (100 mL) and the mixture stirred for 30 min. The precipitate was filtered, washed with water (3 × 30 mL) and dried in vacuo. Compound **8a** was isolated as white crystals (2.49 g, 7.07 mmol, 94.6%). ^1^H NMR (500 MHz, CDCl_3_) δ/ppm 7.09 (s, 2H, H^C3^), 3.92 (t, *J* = 6.5 Hz, 4H, H^a^), 1.89–1.75 (m, 4H, H^b^), 1.06 (t, *J* = 7.4 Hz, 6H, H^c^). This matches the literature data [26]. 

### 3.3. Synthesis of ***8b***

Compound **8a** (1.8 g, 5.11 mmol) and dry Et_2_O (100 mL) were added to a dried flask and cooled to 0 °C using an ice bath. ^n^BuLi (1.6 M in hexanes, 9.58 mL, 15.3 mmol) was added slowly to the solution over a period of 20 min and the temperature maintained at 0 °C for 6 h. Dry DMF (1.19 mL, 15.3 mmol) was added and the solution stirred for 16 h, while warming up to room temperature. The reaction mixture was neutralized with saturated aqueous NH_4_Cl and extracted with CH_2_Cl_2_ (200 mL). The organic phase was dried over MgSO_4_ and concentrated in vacuo. Compound **8b** was isolated as a yellow solid (0.72 g, 2.88 mmol, 56.3%) and used without further purification. ^1^H NMR (500 MHz, CDCl_3_) δ/ppm 10.52 (s, 2H, H^CHO^), 7.43 (s, 2H, H^C3^), 4.05 (t, *J* = 6.5 Hz, 4H, H^a^), 1.93–1.74 (m, 4H, H^b^), 1.06 (m, *J* = 7.4 Hz, 6H, H^c^). This matches the literature data [26].

### 3.4. Synthesis of ***8***

Compound **8b** (0.3 g, 1.2 mmol) was dissolved in EtOH (100 mL), then 4-acetylpyridine (0.54 mL, 0.59 g, 4.8 mmol) and crushed KOH (0.27 g, 4.8 mmol) were added in one portion. Aqueous NH_3_ (32%, 3.1 mL) was added dropwise and the reaction mixture was stirred at room temperature for 16 h. The precipitate was collected by filtration and washed with water (3 × 10 mL), EtOH (3 × 10 mL) and Et_2_O (3 × 10 mL). Compound **8** was isolated as a white solid (0.17 g, 0.26 mmol, 21.6%). Decomp. > 330 °C. ^1^H NMR (500 MHz, CDCl_3_) δ/ppm 8.81 (d, *J* = 6.1 Hz, 8H, H^A2^), 8.12 (s, 4H, H^B3^), 8.11–8.08 (m, 8H, H^A3^), 7.16 (s, 2H, H^C2^), 4.04 (t, *J* = 6.4 Hz, 4H, H^a^), 1.86–1.73 (m, 4H, H^b^), 0.97 (t, *J* = 7.4 Hz, 6H, H^c^). ^13^C{^1^H} NMR (126 MHz, CDCl_3_) δ/ppm 154.5 (C^B2^), 150.4 (C^A2^), 148.0 (C^B4^), 146.0 (C^A4^), 128.9 (C^C1^), 121.3 (C^B3^), 121.0 (C^A3^), 115.0 (C^C2^), 71.1 (C^a^), 22.4 (C^b^), 10.5 (C^c^). C^C3^ was not resolved. ESI-MS *m*/*z* 657.45 [M+H]^+^ (calc. 657.30). High resolution ESI-MS *m*/*z* 657.2983 [M+H]^+^ (calc. 657.2973).

### 3.5. Synthesis of ***9***

2,5-Bis(octoxy)benzene-1,4-dicarbaldehyde (0.15 g, 0.38 mmol) was dissolved in EtOH (40 mL), then 3-acetylpyridine (0.2 g, 1.61 mmol) and crushed KOH (0.108 g, 1.92 mmol) were added to the solution and the reaction mixture was stirred at room temperature for ~16 h. The colourless solution was concentrated in vacuo and left to stand in the freezer at −18 °C for 3 days. Compound **9** precipitated and was isolated as an off-white powder (0.1 g, 0.12 mmol, 31.2%). M.p. = 150.5 °C. ^1^H NMR (500 MHz, CDCl_3_) δ/ppm 9.13 (dd, *J* = 2.3, 0.9 Hz, 4H, H^A2^), 8.74 (dd, *J* = 4.8, 1.7 Hz, 4H, H^A6^), 8.19 (ddd, *J* = 8.0, 2.3, 1.7 Hz, 4H, H^A4^), 7.38 (ddd, *J* = 7.9, 4.8, 0.9 Hz, 4H, H^A5^), 6.69 (s, 2H, H^B3^), 4.17 (p, *J* = 6.8 Hz, 2H, H^x^), 3.85 (t, *J* = 6.6 Hz, 4H, H^a^), 3.5–3.34 (m, 8H, H^y^), 1.77–1.69 (m, 4H, H^b^), 1.42 (m, 4H, H^c^), 1.36–1.19 (m, 16H, H^d/e/f/g^), 0.91–0.75 (m, 6H, H^h^). ^13^C{^1^H} NMR (126 MHz, CDCl_3_) δ/ppm 198.2 (C^C=0^), 153.5 (C^A6^), 150.4 (C^B2^), 149.8 (C^A2^), 135.5 (C^A4^), 132.4 (C^A3^), 129.7 (C^B1^), 123.7 (C^A5^), 113.5 (C^B3^), 68.7 (C^a^), 43.2 (C^y^), 33.5 (C^x^), 31.9 (C^f^), 29.7 (C^b^), 29.5 (C^d/e^), 29.4 (C^d/e^), 26.5 (C^c^), 22.8 (C^g^), 14.3 (C^h^). ESI-MS *m/z* 839.4 [M+H]^+^ (calc. 839.5). IR (solid, ν/cm^−1^) 2953 (w), 2927 (m), 2852 (m), 1681 (s), 1585 (s), 1572 (m), 1511 (m), 1469 (m), 1417 (s), 1379 (m), 1357 (m), 1340 (m), 1285 (m), 1243 (m), 1222 (m), 1200 (s), 1154 (m), 1065 (m), 1040 (w), 1026 (m), 978 (m), 879 (m), 840 (w), 799 (m), 702 (s), 673 (w), 630 (m), 620 (m), 509 (w), 405 (w). Found C 69.41, H 7.11, N 6.43; required for C_52_H_62_N_4_O_6_∙3H_2_O C 69.93, H 7.67, N 6.27. 

### 3.6. {[Co_2_(NCS)_4_(MeOH)_4_(6)_2_]∙2MeOH∙8H_2_O}_n_

A solution of Co(NCS)_2_ (1.75 mg, 0.01 mmol) in MeOH (8 mL) was layered over a solution of **6** (9.76 mg, 0.03 mmol) in CHCl_3_ (5 mL). A few pink crystals of {[Co_2_(NCS)_4_(MeOH)_4_(**6**)_2_]∙2MeOH∙8H_2_O}*_n_* were obtained after 2–4 weeks.

### 3.7. {[Co(NCS)_2_(7)_2_]∙4CHCl_3_}_n_

A solution of Co(NCS)_2_ (1.75 mg, 0.010 mmol) in MeOH (8 mL) was layered over a solution of **7** (4.17 mg, 0.010 mmol) in CHCl_3_ (5 mL). Orange crystals of [Co(NCS)_2_(**7**)_2_∙4CHCl_3_]*_n_* (1.7 mg, 0.0011 mmol, 22% based on **7**) were obtained after 2–4 weeks. The bulk sample was characterized by powder diffraction (see Figure S2).

### 3.8. {[Co(NCS)_2_(8)_2_]·2C_6_H_4_Cl_2_}_n_

A solution of Co(NCS)_2_ (1.75 mg, 0.010 mmol) in MeOH (8 mL) was layered over a solution of **8** (6.57 mg, 0.010 mmol) in 1,2-dichlorobenzene (5 mL). Pink crystals of {[Co(NCS)_2_(**8**)_2_]·2C_6_H_4_Cl_2_}*_n_* (3.9 mg, 0.0035 mmol, 70% based on **8**) were obtained after 2–4 weeks. The bulk sample was characterized by powder diffraction (see Figure S3).

### 3.9. {[Co(NCS)_2_(9)]∙2CHCl_3_}_n_

A solution of Co(NCS)_2_ (0.875 mg, 0.005 mmol) in MeOH (8 mL) was layered over a solution of **9** (12.6 mg, 0.015 mmol) in CHCl_3_ (5 mL). Pink crystals of [Co(NCS)_2_(**9**)∙2CHCl_3_]*_n_* (0.6 mg, 0.00048 mmol, 9.6%) were obtained after 2–4 weeks. The bulk sample was characterized by powder diffraction (see Figure S4).

### 3.10. Crystallography

Single crystal data were collected on a Bruker APEX-II diffractometer; data reduction, solution and refinement used APEX2, SuperFlip and CRYSTALS, respectively [35,36,37]. Structure analysis used Mercury v. 3.7 [38,39]. In {[Co(NCS)_2_(**8**)_2_]·2C_6_H_4_Cl_2_}*_n_*, one 1,2-dichlorobenzene molecule was disordered and was refined isotropically, being treated as a rigid body. SQUEEZE [40] was used to treat the remainder of the solvent region in {[Co(NCS)_2_(**8**)_2_]·2C_6_H_4_Cl_2_}*_n_* and electron density removed equated to one extra molecule of 1,2-dichlorobenzene per formula unit. Powder diffraction data were collected on a Stoe Stadi P powder diffractometer.

{[Co_2_(NCS)_4_(**6**)_2_(MeOH)_4_]·2MeOH∙8H_2_O}*_n_*: C_50_H_70_Co_2_N_14_O_14_S_4_, *M* = 1337.32, pink block, triclinic, space group *P*–1, *a* = 10.7199(6),*b* = 11.3886(7), *c* = 14.7671(11) Å, *α* = 86.374(5), *β* = 85.892(5), *γ* = 88.031(4)°, *U* = 1793.80(13) Å^3^, *Z* = 1, *D_c_* = 1.238 Mg m^−3^, *μ*(Cu-Kα) = 5.235 mm^−1^, *T* = 123 K. Total 24308 reflections, 6218 unique, *R*_int_ = 0.051. Refinement of 3947 reflections (364 parameters) with *I* > 2σ (*I*) converged at final *R*1 = 0.1347 (*R*1 all data = 0.1693), *wR*2 = 0.3635 (*wR*2 all data =0.3934), gof = 1.0474. CCDC 1550589.

{[Co(NCS)_2_(**7**)_2_]·4CHCl_3_}*_n_*: C_56_H_42_Cl_12_CoFe_2_N_8_S_2_, *M* = 1487.19, orange block, monoclinic, space group *P*2_1_/*n*, *a* = 10.5796(7),*b* = 17.4860(12), *c* = 16.9126(11) Å, *β* = 99.047(3)°, *U* = 3089.8(4) Å^3^, *Z* = 2, *D_c_* = 1.598 Mg m^−3^, *μ*(Cu-Kα) = 11.565 mm^−1^, *T* = 123 K. Total 21236 reflections, 5706 unique, *R*_int_ = 0.030. Refinement of 5555 reflections (367 parameters) with *I* > 2σ (*I*) converged at final *R*1 = 0.0473 (*R*1 all data = 0.0483), *wR*2 = 0.0964 (*wR*2 all data =0.0966), gof = 0.9222. CCDC 1550588.

{[Co(NCS)_2_(**8**)_2_]·2C_6_H_4_Cl_2_}*_n_*: C_56_H_44_Cl_4_CoN_8_O_2_S_2_, *M* = 1125.89, pink block, monoclinic, space group *P*2_1_/*c*, *a* = 10.2136(9),*b* = 19.3452(17), *c* = 16.2214(15) Å, *β* = 107.027(3)°, *U* = 3064.6(5) Å^3^, *Z* = 2, *D_c_* = 1.22 Mg m^−3^, *μ*(Cu-Kα) = 4.787 mm^−1^, *T* = 123 K. Total 20265 reflections, 5638 unique, *R*_int_ = 0.029. Refinement of 4472 reflections (298 parameters) with *I* > 2σ (*I*) converged at final *R*1 = 0.1258 (*R*1 all data = 0.1287), *wR*2 = 0.1436 (*wR*2 all data =0.1437), gof = 0.9942. CCDC 1550587.

{[Co(NCS)_2_(**9**)]·2CHCl_3_}*_n_*: C_56_H_64_Cl_6_CoN_6_O_6_S_2_, *M* = 1252.94, pink block, triclinic, space group *P*–1, *a* = 9.5196(10),*b* = 11.1662(13), *c* = 14.4682(16) Å, *α* = 80.088(6), *β* = 78.780(5), *γ* = 86.642(6)°, *U* = 1485.5(3) Å^3^, *Z* = 1, *D_c_* = 1.400 Mg m^−3^, *μ*(Cu-Kα) = 5.838 mm^−1^, *T* = 123 K. Total 18364 reflections, 5368 unique, *R*_int_ = 0.049. Refinement of 4997 reflections (349 parameters) with *I* >2σ (*I*) converged at final *R*1 = 0.1210 (*R*1 all data = 0.1256), *wR*2 = 0.2862 (*wR*2 all data =0.2891), gof = 0.9924. CCDC 1550586.

## 4. Conclusions

In conclusion, we have described coordination polymer and network assemblies that result from crystal growth under ambient conditions when Co(NCS)_2_ is combined with monotopic ligands **6** and **7**, and ditopic ligands **8** and **9**. Although ligand **6** contains both pyrimidine and 4,2’:6’,4”-tpy metal-binding domains, it binds cobalt only through the outer *N*-donors of the 4,2’:6’,4”-tpy unit. In **7**, the ferrocenyl functionality is coordinatively innocent and, like **6**, ligand **7** acts as a V-shaped linker. {[Co(NCS)_2_(**7**)_2_]∙4CHCl_3_}*_n_* contains a (4,4) net defined by 4-connecting cobalt nodes. In contrast, coordinated MeOH (a solvent used in all the experiments reported here) in {[Co_2_(NCS)_4_(MeOH)_4_(**6**)_2_]∙2MeOH∙8H_2_O}*_n_* blocks two of the octahedral sites and the resulting assembly is a 1D-coordination polymer. This result highlights the role of serendipity in the assembly process. On the other hand, inter-sheet π-stacking interactions between ferrocenyl and pyridyl units in {[Co(NCS)_2_(**7**)_2_]∙4CHCl_3_}*_n_* may assist in directing this assembly.

By moving to the ditopic ligand **8** which may adopt limiting 4-connecting planar or approximately tetrahedral nodal geometries, it is possible to realize a 3D-framework. {[Co(NCS)_2_(**8**)_2_] 2C_6_H_4_Cl_2_}*_n_* consists of a {6^5^.8} **cds** net which is defined by a combination of metal and ligand planar 4-connecting nodes. This contrasts with the 3D {4^2^.8^4^} **lvt** net present in [Co(NCS)_2_(**3**)·4CHCl_3_]*_n_* where the metal and ligand act as 4-connecting planar and approximately tetrahedral nodes, respectively [16]. On going from **3** to **9**, a more flexible backbone is introduced to the ligand, and reaction of **9** with Co(NCS)_2_ leads to {[Co(NCS)_2_(**9**)]∙2CHCl_3_}*_n_* which contains a (4,4) net defined by both metal and ligand planar 4-connecting nodes. The *^n^*octoxy tails of **9** protrude from each side of the (4,4) net and thread through adjacent sheets; the arene-attached *^n^*octoxy chains associate through a combination of van der Waals and C–H...π interactions.

Taking Co(NCS)_2_ as a common building block with a preference for octahedral coordination, this investigation has once again [24,41] illustrated the difficulties of predicting and understanding coordination assembly algorithms, even when the ligand has well-defined directional properties, in particular in assembly environments containing potentially coordinating solvent molecules.

## Supplementary Materials

The following are available online at www.mdpi.com/1996-1944/10/7/728/s1. Supplementary information contains Figure S1: HMQC spectrum of ligand 9. Figures S2–S4: Comparisons of powder diffraction patterns for bulk samples and predicted patterns from single crystal diffraction; Figure S5: Comparison of the conformations of coordinated ligands **3** and **9** in {[Co(NCS)_2_(**3**)]∙4CHCl_3_}*_n_* and {[Co(NCS)_2_(**9**)]∙2CHCl_3_}*_n_*.
