# Supplementary material for: 4,2’:6’,4”- and 3,2’:6’,3”-Terpyridines: The Conflict between Well-Defined Vectorial Properties and Serendipity in the Assembly of 1D-, 2D- and 3D-Architectures
Source: Materials (Basel). 2017 Jun 30;10(7):728. doi: 10.3390/ma10070728 (PMC5551771; doi:10.3390/ma10070728)
Supplement: Supplementary file 1 [file materials-10-00728-s001.pdf]

Supplementary information to accompany:

4,2':6',4''- and 3,2':6',3''-terpyridines: the conflict between well-defined vectorial properties and serendipity in the assembly of 1D-, 2D- and 3D-architectures

Y. Maximilian Klein[a], Alessandro Prescimone[a], Edwin C. Constable[a] and Catherine E. Housecroft*[a]

[a]Department of Chemistry, University of Basel, Spitalstrasse 51, 4056 Basel, Switzerland.

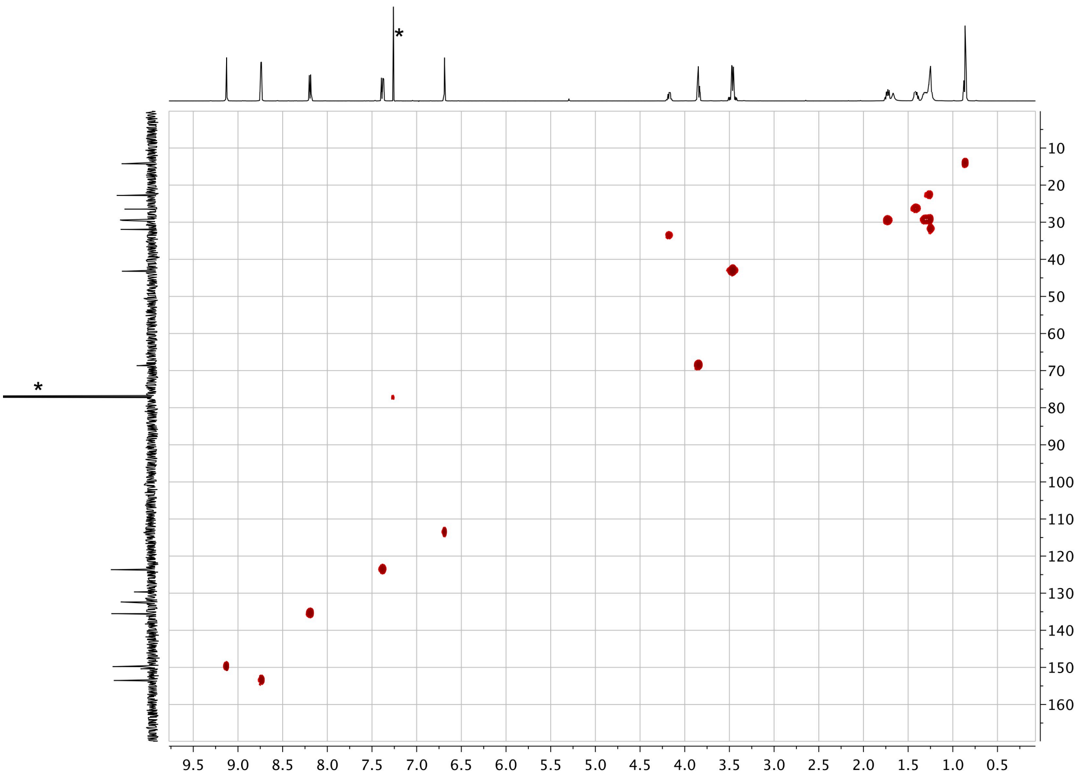

Fig. S1. HMQC (500 MHz ^{1}H NMR and 126 MHz ^{13}C NMR) spectrum of compound **9** in $CDCl_3$. * = residual $CHCl_3$.

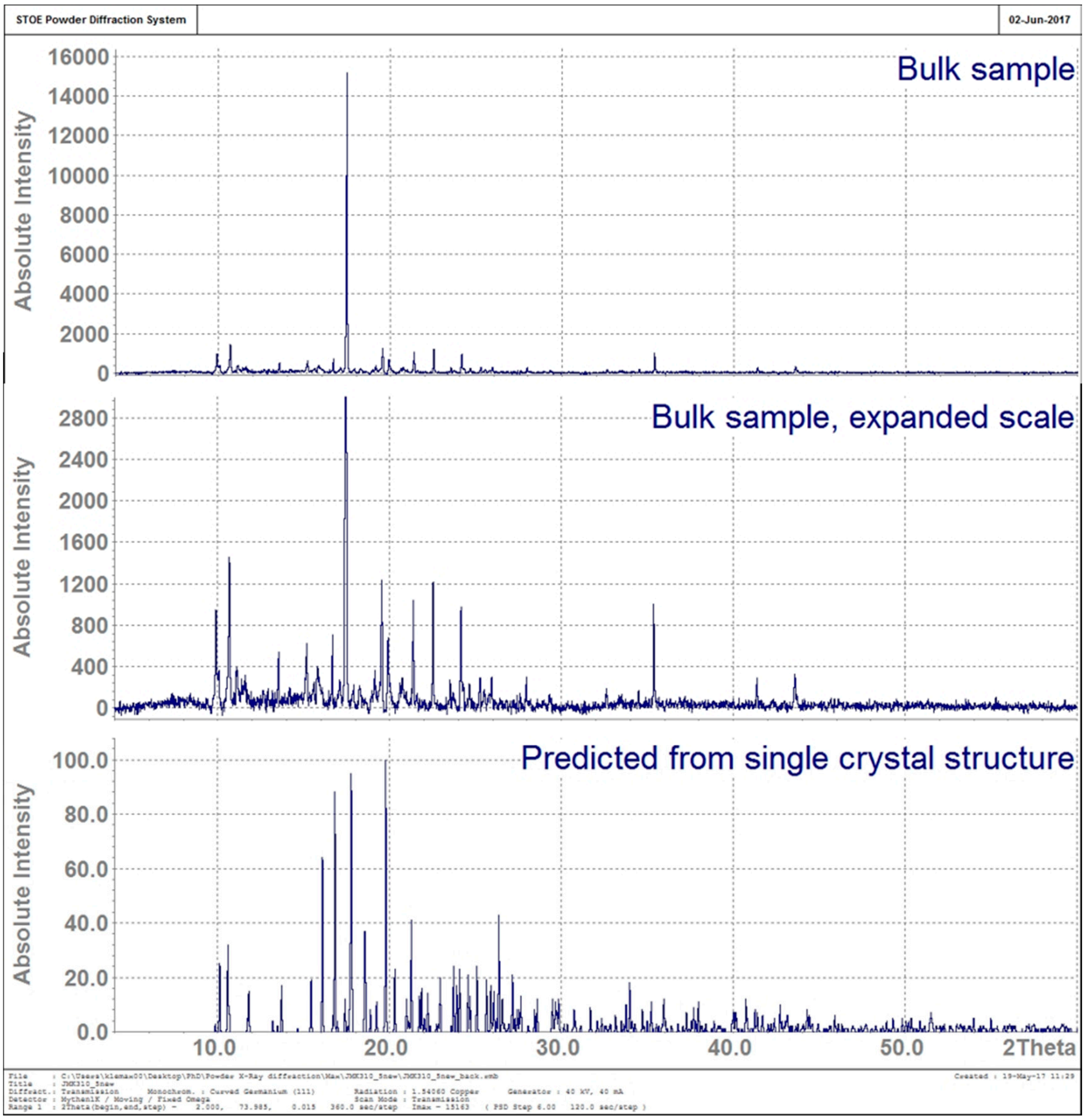

Fig. S2. Comparison of the powder diffraction patterns of the bulk sample obtained from the reaction of $Co(NCS)_2$ and **7** (top two patterns, room temperature) and that predicted from the single crystal structure of $\{[Co(NCS)_2(\mathbf{7})_2]\cdot4CHCl_3\}_n$ (123 K). Differences between the peak intensities of the measured bulk samples compared to predicted patterns most likely arise from preferred orientations of the crystals in the bulk material. Grinding the sample more did not change the relative peak intensities but signal-to-noise ratios decreased dramatically.

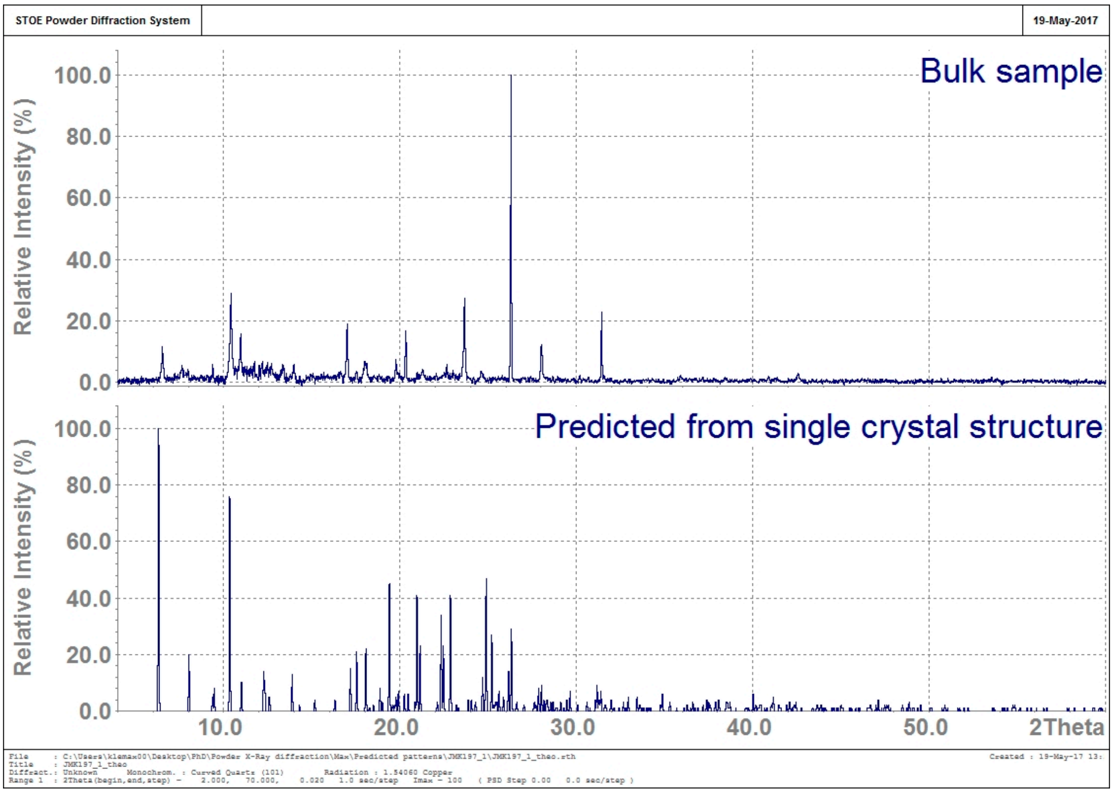

Fig. S3. Comparison of the powder diffraction patterns of the bulk sample obtained from the reaction of $Co(NCS)_2$ and **8** (top, room temperature) and that predicted from the single crystal structure of $\{[Co(NCS)_2(\mathbf{8})_2]\cdot 2C_6H_4Cl_2\}_n$ (123 K).

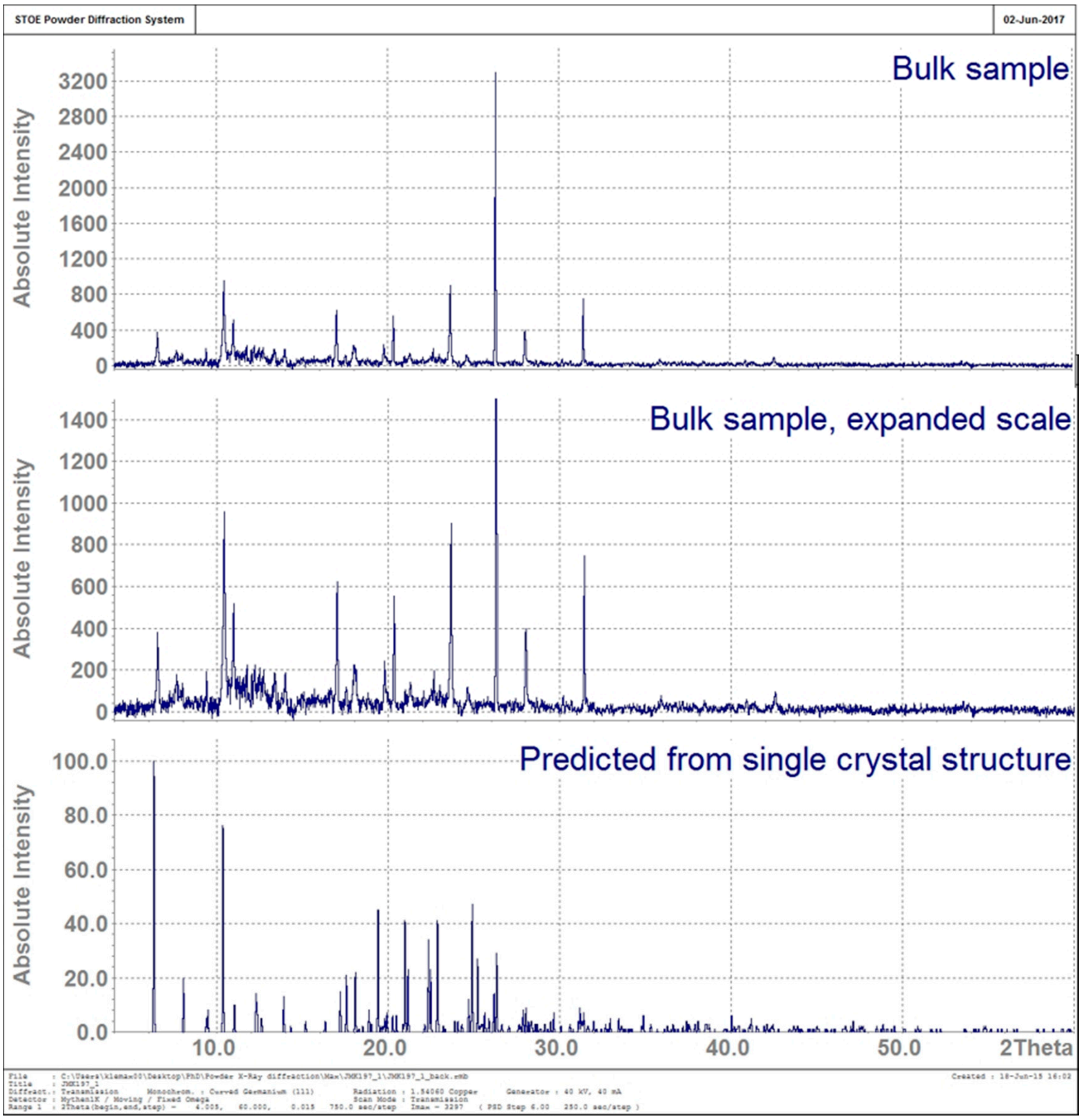

Fig. S4. Comparison of the powder diffraction patterns of the bulk sample obtained from the reaction of Co(NCS)$_2$ and **9** (top, room temperature) and that predicted from the single crystal structure of {[Co(NCS)$_2$(**9**)]·2CHCl$_3$}$_n$ (123 K). Differences between the peak intensities of the measured bulk samples compared to predicted patterns most likely arise from preferred orientations of the crystals in the bulk material. Grinding the sample more did not change the relative peak intensities but signal-to-noise ratios decreased dramatically.

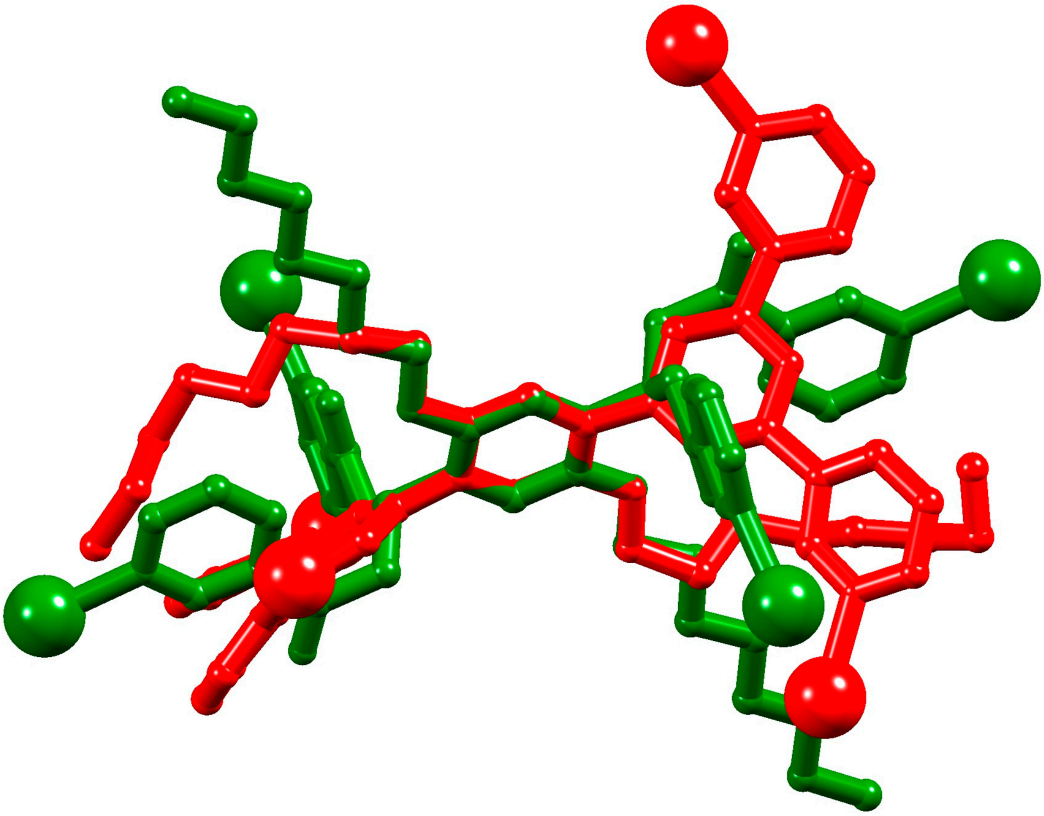

Fig. S5. Comparison of the conformations of the coordinated ligands **3** (red) and **9** (green) in $\{[Co(NCS)_2(\mathbf{3})]\cdot 4CHCl_3\}_n$ and $\{[Co(NCS)_2(\mathbf{9})]\cdot 2CHCl_3\}_n$, respectively. The central arene rings have been overlaid. Cobalt atoms are shown as large spheres. Ligand **3** acts as an approximately tetrahedral 4-connecting node, and **9** as a planar 4-connecting node.
